# A Phase II Clinical Trial of CPI-613 in Patients with Relapsed or Refractory Small Cell Lung Carcinoma

**DOI:** 10.1371/journal.pone.0164244

**Published:** 2016-10-12

**Authors:** Thomas W. Lycan, Timothy S. Pardee, William J. Petty, Marcelo Bonomi, Angela Alistar, Zanetta S. Lamar, Scott Isom, Michael D. Chan, Antonius A. Miller, Jimmy Ruiz

**Affiliations:** 1 Department of Medicine, Section on Hematology and Oncology, Wake Forest School of Medicine, Winston-Salem, NC, United States of America; 2 Department of Biostatistical Sciences, Wake Forest School of Medicine, Winston-Salem, NC, United States of America; 3 Department of Radiation Oncology, Wake Forest School of Medicine, Winston-Salem, NC, United States of America; 4 W.G. (Bill) Hefner Veteran Administration Medical Center, Cancer Center, Salisbury, NC, United States of America; Tata Memorial Centre, INDIA

## Abstract

**Background:**

Small cell lung cancer (SCLC) is a common lung cancer which presents with extensive stage disease at time of diagnosis in two-thirds of patients. For treatment of advanced disease, traditional platinum doublet chemotherapy induces response rates up to 80% but with few durable responses. CPI-613 is a novel anti-cancer agent that selectively inhibits the altered form of mitochondrial energy metabolism in tumor cells.

**Methods:**

We evaluated CPI-613 with a single-arm, open-label phase II study in patients with relapsed or refractory SCLC. CPI-613 was given at a dose of 3,000 mg/m2 on days 1 and 4 of weeks 1–3 of 4 week cycle. The primary outcome was response rate as assessed by CT imaging using RECIST v1.1 criteria. Secondary outcomes were progression-free survival (PFS), overall survival (OS), and toxicity. Twelve patients were accrued (median age 57yo) who had previously received between 1 and 4 lines of chemotherapy (median 1) for SCLC with a treatment-free interval of less than 60 days in 9 of the 12 patients.

**Results:**

No complete or partial responses were seen. Ten patients (83%) progressed as best response and 2 (17%) were not evaluable for response. Median time to progression was 1.7 months (range 0.7 to 1.8 months). Eleven patients (92%) died with median overall survival of 4.3 months (range 1.2 to 18.2 months). The study was closed early due to lack of efficacy. Of note, three out of three patients who progressed after CPI-613 and were subsequently treated with standard topotecan then demonstrated treatment response with survival for 18.2, 7.4, and 5.1 months. We conducted laboratory studies which found synergy in-vitro for CPI-613 with topotecan.

**Conclusions:**

Single agent CPI-613 had no efficacy in this study. Further study of CPI 613 in combination with a topoisomerase inhibitor is warranted.

## 1. Introduction

Small cell lung cancer (SCLC) is an aggressive neuroendocrine carcinoma where two-thirds of patients present with extensive stage disease at time of diagnosis. First-line treatment with traditional platinum doublet chemotherapy has a high response rate of 85% but with a limited median duration of response of 7 months for limited disease and 5.5 months for extensive disease [1]. Relapsed SCLC has a very poor prognosis. O’Brien et al [2] found that 70 patients with relapsed SCLC managed with best supportive care had a median survival of 3.5 months and a six-month survival rate of 26%. Even among patients with limited stage disease, the long-term prognosis remains poor with 10% overall survival at 5 years.

Pyruvate dehydrogenase (PDH) is a key mitochondrial enzyme that acts as the entry point for pyruvate into the tricarboxylic acid (TCA) cycle, generating cellular energy and biosynthetic precursors. PDH is regulated by PDH regulatory kinases (PDKs) and its activity is substantially altered in cancer cells. Classically, the Warburg effect describes how cancer cells preferentially generate energy by shunting pyruvate away from PDH and the aerobic TCA cycle and instead to lactate dehydrogenase (LDH) and anaerobic glycolysis. However, cancer cells still require an influx of TCA intermediaries via PDH for the biosynthesis of lipids, proteins, and nucleic acids [3]. Therefore, the activity of mitochondrial PDH remains a necessary component of malignant cell function. Lipoate is a necessary catalytic cofactor for PDH and multiple other mitochondrial enzymes.

CPI-613 is the first agent from a novel class of lipoate analogs which were developed as potential anticancer drugs. Structurally similar to lipoate, CPI-613 activates lipoate-sensitive PDKs which phosphorylate three specific phosphoserines on LDH. This phosphorylation inhibits LDH which suppresses mitochondrial ATP production and leads to cell death [4]. Since mitochondrial function is necessary for malignant growth across a wide range of tumor types, CPI-613 exhibits anti-cancer activities against various types of solid and hematologic malignancies, including small cell lung cancer (NCI-H69 cell line). And given its novel mechanism of action against mitochondrial function, CPI-613 is unlikely to have any cross-resistance to traditional chemotherapeutic agents used in SCLC. Phase I dose-escalation trials of CPI-613 have found it to be well-tolerated as a two-hour intravenous infusion at doses up to 3,000 mg/m2 [5]. The primary objective of this phase II study was to evaluate the response rate (RR) of CPI-613 in patients with relapsed or refractory SCLC who have failed 1st line chemotherapy. Secondary objectives were to evaluate the safety, toxicity, progression-free-survival (PFS) and overall survival (OS) of CPI-613 in these patients.

## 2. Materials and Methods

### 2.1 Patient selection

Patients with either limited or extensive stage SCLC which had relapsed or been refractory to at least one line of chemotherapy were eligible for this study. Other eligibility criteria included age ≥18 years; performance status (Eastern Cooperative Oncology Group scale) of 0 to 2; platelet count ≥100,000 cells/mm3, absolute neutrophil count [ANC] ≥1500 cells/mm3; aspartate aminotransferase [AST] ≤3x upper normal limit [UNL]; bilirubin ≤1.5x UNL; serum creatinine ≥1.5 mg/dL; albumin >3.0 g/dL; and double-lumen central venous access. Exclusion criteria included life expectancy less than 3 months; untreated central nervous system (CNS) or epidural metastases; active heart disease; or active infection including HIV. All patients had measurable evidence of tumor progression or recurrence on CT imaging performed within 2 weeks of starting treatment. Patients were required to have a complete recovery from the toxicity of prior therapy. This single institution study was approved by the Wake Forest Health Sciences Institutional Review Board and monitored by the Comprehensive Cancer Center’s Safety and Toxicity Review Committee. Written and informed consent was obtained for all subjects in accordance with the Declaration of Helsinki. The trial was registered at clinicaltrials.gov (Identifier: NCT01931787).

### 2.2 Treatment

**Fig 1:**
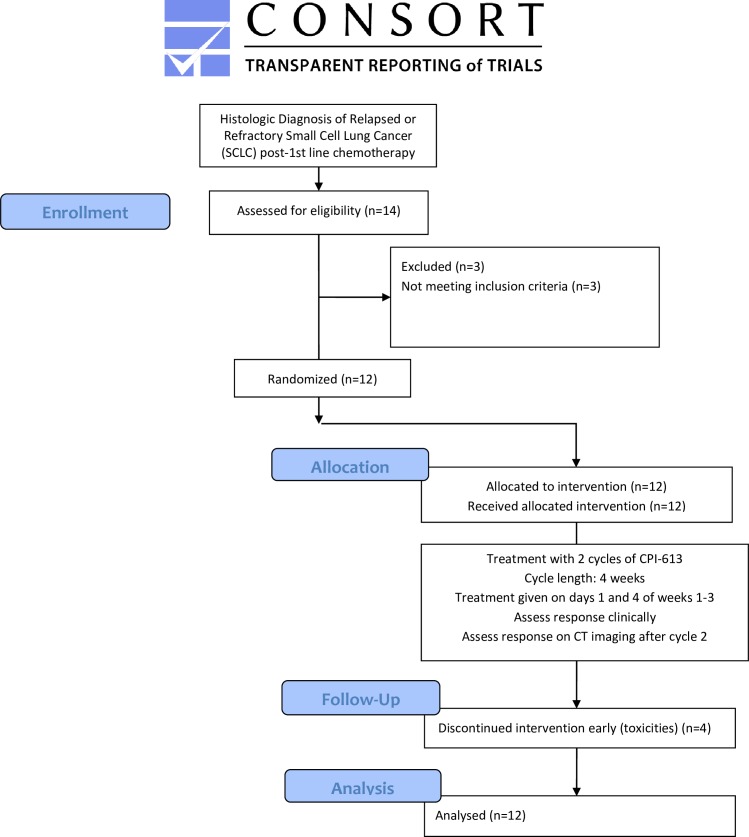
Flow diagram of study protocol.

We evaluated CPI-613 with a single-arm, open-label phase II study (Fig 1) in patients with relapsed or refractory SCLC. CPI-613 was given at a dose of 3,000 mg/m2 IV on days 1 and 4 of weeks 1–3 of 4 week cycle. Two cycles were given to complete a treatment course prior to re-evaluation.

### 2.3 Dose delays and modifications

No prophylactic treatments for drug-related symptoms were planned to assess for toxicity. Once a participant became symptomatic then supportive treatment could be prescribed. CPI-613 therapy was delayed for any grade 2–4 toxicities (other than nausea) and could only be resumed once toxicities had improved to grade 1 or below. For grade 3–4 toxicities, subsequent doses were reduced by 50%. Acute kidney injury suspected to be due to CPI-613 had dose reductions of 15% for grade 1, 25% for grade 2, and 50% for grades 3/4. Blood transfusions were given as needed in order to maintain hemoglobin ≥8 g/dL during the course of the study.

### 2.4 Patient assessments

Evaluation of symptoms, performance status, blood counts, kidney/liver function, lactate dehydrogenase (LDH) was done at baseline and on day 1 of weeks 1, 2, and 3 of each treatment cycle. Patients were reevaluated with history, physical, and laboratory studies at the completion of each 4 week cycle. Tumor assessments were conducted by imaging with contrast CT of the thorax and abdomen and/or by PET/CT at baseline and after every two cycles. Efficacy was determined by tumor size comparison on imaging using the Response Evaluation Criteria in Solid Tumors (RECIST, v1.1) [6]. All patients were followed until death for disease-free and overall survival endpoints. Toxicity was described as per version 4.0 of the NCI Common Toxicity Criteria (CTC) for Toxicity and Adverse Event Reporting. After treatment termination, clinical information was collected bimonthly in follow-up.

### 2.5 Statistical methods

Efficacy assessments were based on all evaluable patients. As a single arm pilot study with twenty patients targeted for accrual, the primary goal was to gather preliminary data on response rates. The primary outcome was response rate (RR) as assessed by CT imaging. Secondary outcomes were progression-free survival (PFS), overall survival (OS), and toxicity. PFS and OS were determined from the first dose of CPI-613. Since none of the first 12 patients showed evidence of tumor response (95% exact confidence interval for response rate 0–26.5%) with multiple toxicity events, the study was stopped early for futility. Descriptive analysis alone was used for all end points (S1 File and S2 File).

## 3. In Vitro Methods

### 3.1 Cell culture and viability assays

Human lines were obtained from the ATCC and maintained in RPMI media (Gibco) supplemented with 10% FBS, penicillin and streptomycin. Viability assays were carried out according to the manufacturer’s protocols with the Cell Titer-Glo system (Promega).

### 3.2 Statistical analysis

Groups of 3 or more were analyzed using a one way ANOVA. All means were compared by a Student’s 2-tailed t test. All analyses were performed using GraphPad Prism Version 5.02 (GraphPad Software). A p value ≤ 0.05 was considered significant. Synergy was determined using the method of Chou and Talay [7]. All combinatorial indices were calculated using CalcuSyn Version 2.11 (Biosoft).

## 4. Results

### 4.1 Patients

A total of 12 patients (8 men) with recurrent or refractory SCLC were enrolled (median age 57 years, range 32–73) with accrual from October 2013 to September 2015. Three patients did not meet inclusion criteria due to active infection, cytopenias, or absence of venous access. Patient characteristics are listed in Table 1. Initial extent of enrolled patients included 8 with extensive stage disease and 4 with limited stage disease. Ten patients had an ECOG performance status (PS) of 1 or less, with two patients having a PS of 2. Five patients had previously received thoracic radiation and chemotherapy. Patients in this trial had received between 1 and 4 lines of chemotherapy (median 1) prior to enrollment. Less than half of the participants (42%) in this study had platinum resistant disease as evidenced by a treatment-free interval of less than 3 months due to progression of disease after completing first-line chemotherapy. Several of the participants (25%) received several prior lines of chemotherapy before enrollment in this study. First line therapy for all patients was with etoposide (80–100 mg/m2) and either carboplatin (AUC 5) or cisplatin (80 mg/m2). Three patients received more than 1 line of chemotherapy before study enrollment. Two participants with chemotherapy-sensitive disease received a repetition of first-line platinum-based therapy as subsequent therapy before enrolling with this study. One other participant with chemotherapy-resistant disease was started on subsequent lines of chemotherapy with topotecan, followed by docetaxel, and then gemcitabine prior to enrollment.

**Table 1:** Patient characteristics.

		N		%
Evaluable		12		
Non-evaluable (excluded from analysis)		3		
Age (yr)	Range	32–73		
	Median	57		
Gender	Male	8	67%	
	Female	4	33%	
Race	Caucasian	10	83%	
	African-American	2	17%	
Performance status (ECOG)	0	1	8%	
	1	9	75%	
	2	2	17%	
Initial staging	Limited	4	33%	
	Extensive, no brain metastases	4	33%	
	Extensive, with brain metastases	4	33%	
			
Prior radiation treatment to chest	Yes	5	42%	
	No	7	58%	
Previous lines of chemotherapy	1	9	75%	
	>1	3	25%	
	Range	1–4		
Treatment-free interval (days)	0- <30	4	33%	
	30- <60	1	8%	
	60- <90	0	0%	
	> = 90	7	58%	
	Average	97		
	Median	97		
	SD	83.5		

Following enrollment on this trial, patients in total received a total of 16.3 cycles (median 1.63) of therapy with a median maximum cumulative CPI dosage of 30,000 mg/m2 (range 9,000 to 36,000). Three participants were excluded from analysis; 1 had an infected Port-A-Cath and refused replacement of central venous access; 1 had a worsening pneumonia within days of starting treatment which was felt to be unrelated to treatment; and 1 had worsening cytopenias on the day he was to start the study which did not improve.

### 4.2 Treatment efficacy

**Fig 2:**
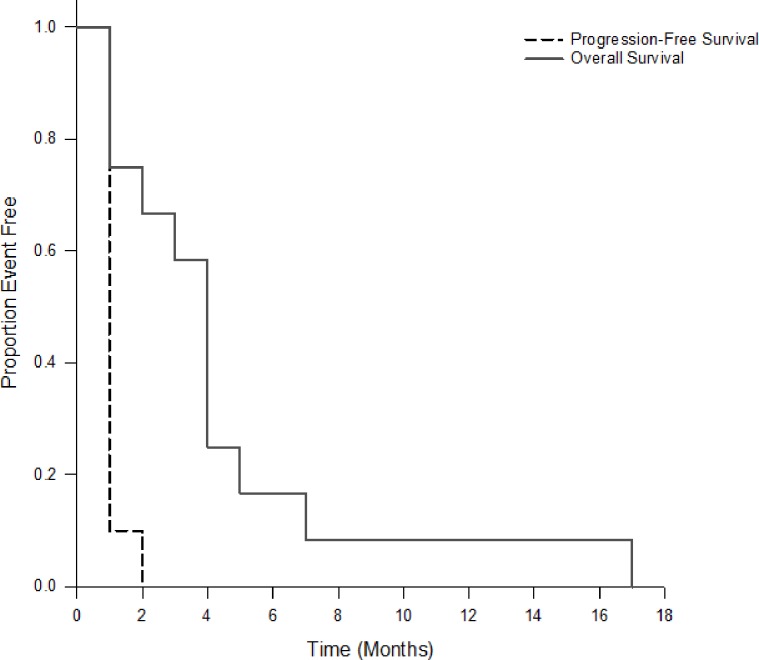
Kaplan-Meier curve of overall survival and progression-free survival.

No complete or partial responses were seen. Ten patients (83%) progressed as best response and 2 (17%) were not evaluated for response by imaging. Median time to progression or else death from any cause was 1.7 months (range 0.7 to 2.8 months). All twelve patients died with median overall survival of 4.3 months (range 1.2 to 18.2 months) and no patients were lost to follow-up (Fig 2).

**Fig 3:**
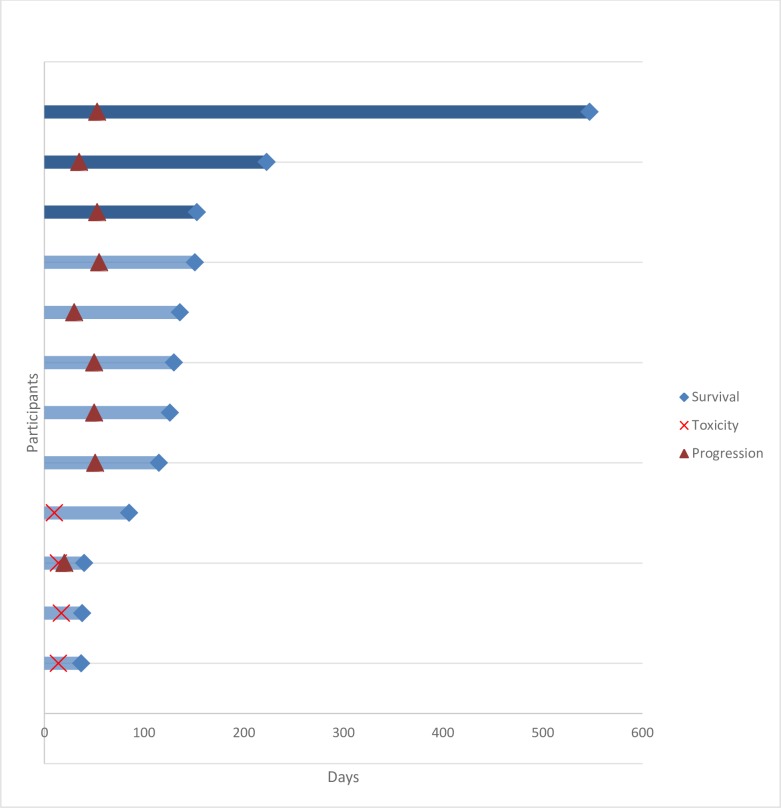
Swimmer’s plot. Swimmer’s plot of time to progression, time to toxicity causing early discontinuation of study drug, and time to death (overall survival). Three of three patients who progressed on CPI-613 and were subsequently started on topotecan then demonstrated treatment response with survival for 547, 223, and 153 days (dark blue rows).

Three of three patients who progressed on CPI-613 and were subsequently started on topotecan then demonstrated treatment response with survival for 18.2, 7.4, and 5.1 months (Fig 3). At time of progression on CPI-613, two of these three patients were stable clinically but had progression evident on CT imaging alone–seen as increased size of pre-existing thoracic disease as well as new lesions (one had a new 1.2cm adrenal nodule; the other had new hepatic nodules). The third patient had progression clinically (worsening abdominal pain and nausea) and on imaging (increased size of thoracoabdominal disease and new retroperitoneal lymphadenopathy).

### 4.3 Toxicity

Four patients had discontinuation of study drug early due to toxicities and three of those four due to toxicities which were at least partially due to study drug. The most common toxicities that were related or probably related to study drug were nausea (58%) and vomiting (42%); all others are described in Table 2. One patient had persistence of grade 3 headaches associated with previous radiation treatments and then developed new onset of nausea, vomiting, and fatigue (each grade 2); study drug was discontinued and he opted for hospice. The second patient developed falls, nausea, and fatigue (each grade 2) so study drug was discontinued. The third patient had anorexia, nausea, and vomiting (each grade 2) contributing to dehydration (grade 3) and worsening chronic hyponatremia from malignant SIADH; study drug was discontinued. A fourth patient had unrelated toxicity; he had worsening of prior headaches (grade 3) which were found to be due to new ocular metastases; he declined further chemotherapy and study drug was discontinued. Several other serious adverse events occurred during this trial which were not felt to be related to the study drug. One patient was hospitalized for a pulmonary embolism and one month later was hospitalized again for obstructive jaundice due to pancreatic metastases. Another patient was hospitalized for acute pancreatitis due to pancreatic metastases. One patient had recurrence of chronic atypical chest pain after the study drug infusion which was relieved by ranitidine. One patient was admitted for a GI bleed two weeks after receiving the last dose of study drug. One patient had one of their CPI-613 treatments delayed due to dysfunction of their Port-A-Cath.

**Table 2:** Summary of toxicities with related or probable attribution.

	# Participants by CTC Toxicity Grade				
Toxicity	Grade 2	Grade 3	Grade 4	Grade 5	Total
Anemia	0	0	1	0	1
Dysgeusia	1	0	0	0	1
Fall	1	0	0	0	1
Hypocalcemia	1	0	0	0	1
Hyponatremia	0	1	0	0	1
Lymphopenia	0	0	1	0	1
Nausea	7	0	0	0	7
Vomiting	5	0	0	0	5

### 4.4 In vitro results

**Fig 4:**
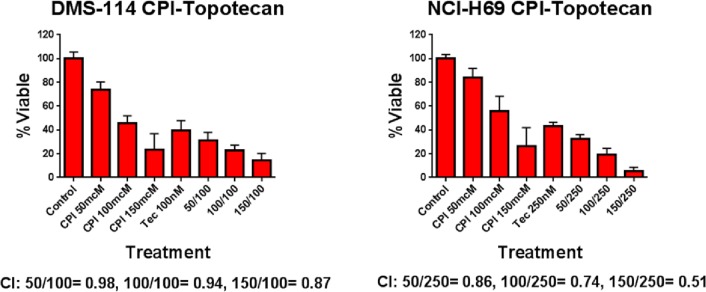
In vitro assessment of the combination of CPI-613 with topotecan. DMS-114 and NCI-H69 SCLC cell lines were exposed to the indicated drug for 72 hours and then cell viability was assessed. Combinatorial indices were calculated for the indicated drug combinations. CI = Combinatorial Index, CPI = CPI-613, Tec = Topotecan.

Given the high rate of response seen in patients who went on to topotecan salvage therapy, we assessed the ability of CPI-613 to synergize with topotecan in vitro. CPI-613 and topotecan were cytotoxic at multiple doses. Combinatorial indices were calculated and demonstrated synergy for the combination (Fig 4). These data suggest that CPI-613 may be effective when combined with topotecan for patients with relapsed SCLC.

## 5. Discussion

This was an open-label, single institution study of the novel agent CPI-613 in patients with histologically proven recurrent or refractory SCLC. The results of this study suggest that CPI-613 monotherapy at the dose and schedule tested in this trial was not efficacious in disease response.

CPI-613 was selected for evaluation in SCLC patients based on previous early phase studies in both solid and hematologic malignancies that showed minimal toxicities. The first was a dose-escalation phase I clinical trial of monotherapy CPI-613 (NCT00741403) which enrolled patients with a variety of advanced malignancies; several case reports from that study provided examples of clinical efficacy at varying doses. Lee et al [8] described an 88-year-old female with a widely metastatic pancreatic neuroendocrine tumor whose disease stabilized on CPI-613 for over one year with minimal toxicity. Senzer et al [9] described a 73-year-old male with relapsed stage IV metastatic hepatocellular carcinoma who had progression-free survival of 10 months on CPI-613 with minimal grade 3–4 toxicities. The next phase I study was by Pardee et al. [5] which utilized single agent CPI-613 in 26 heavily treated relapsed or refractory hematologic malignancies. There were 21 evaluable patients and 4 achieved an objective response and 2 had prolonged control of disease. Overall, CPI-613 was well tolerated and Its standard dosing and schedule was adopted from this study and based on the maximum tolerated dose of 2,940 mg/m2 over 2 hours on days 1 and 4.

Combination therapy of CPI-613 with cytotoxic chemotherapy has shown promise in both solid and hematologic malignancies. Relapsed or refractory AML patients treated with the combination of CPI-613 (2,500 mg/m2) with high-dose cytarabine (HDAC; 1,500–3,000 mg/m2) and mitoxantrone (6 mg/m2) found a satisfactory response rate of 42% across all 65 evaluable patients; a subset of patients with poor risk cytogenetics had a similar response rate (47%). This study found that the most common toxicities attributed to CPI-613 were diarrhea and nausea, mostly grade 1 or 2. The final results of this study are pending for the combination of CPI-613 with HDAC and mitoxantrone for relapsed or refractory AML [10]. A pilot study of 6 patients with stage IV pancreatic cancer found that the combination of CPI-613 (70–320 mg/m2) with gemcitabine (1,000 mg/m2) was well-tolerated and that prolonged survival was associated with increased doses of CPI-613 among the 3 treatment-naïve patients [11]. A recent abstract from a phase I clinical trial of patients with stage IV pancreatic cancer assessed the addition of CPI-613 to the modified FOLFIRINOX regimen as first line therapy. That study of 21 enrolled patients found the maximum tolerated dose of CPI-613 to be 500 mg/m2 without any additional toxicity. In the preliminary assessment of efficacy, the addition of CPI-613 led to a higher response rate (53.9% as compared to 31.6% reported with FOLFIRINOX alone) including one complete response and two near-complete responses; a larger phase II study is currently being planned based on these results [12].

Although our clinical trial with single agent CPI-613 did not yield a significant signal for treatment response, 3 of 3 patients who went on to topotecan had a positive response. Relapsed or refractory SCLC has a low partial response rate of up to 24% in patients treated with topotecan, the only FDA-approved chemotherapy in this setting [2,13]. Topotecan use as standard care has been shown to have a modest prolongation of median survival from 3.5 months to 6.5 months [2]. In our study, patients who went on to topotecan treatment had an OS of 18.2, 7.4, and 5.1 months, respectively. Previous clinical trials combining CPI-613 with traditional chemotherapy in other malignancies have shown impressive response rates as noted above. In terms of mechanism, impairment of mitochondrial function may limit malignant cells ability to counteract the effects of chemotherapy and increase response rates. Therefore, we questioned whether CPI-613 served as a table-setter for subsequent treatment with cytoxic chemotherapy. Our in vitro assessment of the combination of CPI-613 and topotecan when exposed to two SCLC cell lines found a potentially synergistic effect (Fig 2).

## 6. Conclusions

Monotherapy CPI-613 at the dose and schedule tested in this trial was not efficacious in terms of disease response. Based on patients who went on to have a response to topotecan after the study and on our in vitro data, we believe that a novel combination of CPI-613 and topotecan may have clinical benefit in this difficult to treatment malignancy. Further study of CPI 613 in combination with a topoisomerase inhibitor in patients is warranted.

## Supporting Information

S1 File TREND statement checklist. (PDF)

S2 File Study protocol. (DOCX)
