# Supplementary material for: A Phase II Clinical Trial of CPI-613 in Patients with Relapsed or Refractory Small Cell Lung Carcinoma
Source: PLoS One. 2016 Oct 12;11(10):e0164244. doi: 10.1371/journal.pone.0164244 (PMC5061374; doi:10.1371/journal.pone.0164244)
Supplement: S2 File — (DOCX) [file pone.0164244.s002.docx]

Principal Investigator: Jimmy Ruiz, MD

Department of Internal Medicine

Section on Hematology and Oncology

Wake Forest School of Medicine

Medical Center Boulevard

Winston-Salem, NC 27157

[jruiz@wakehealth.edu](mailto:jruiz@wakehealth.edu)

336-716-4464

Co-Investigators: William Jeff Petty, MD

Wake Forest School of Medicine

Section on Hematology and Oncology

wpetty[@wakehealth.edu](mailto:ablackst@wakehealth.edu)

336-716-4977

Antonius Miller, MD

Wake Forest School of Medicine

Section on Hematology and Oncology

aamiller@wakehealth.edu

336-716-4464

Timothy Pardee, MD, PhD

Wake Forest School of Medicine

Section on Hematology and Oncology

[tspardee@wakehealth.edu](mailto:tspardee@wakehealth.edu)

336-716-4464

Biostatistician: Ralph B. D’Agostino, Jr., PhD

Comprehensive Cancer Center of Wake Forest University

rdagosti@wakehealth.edu

336-716-9011

Research RN: Sharon Averill, RN, CRA

Comprehensive Cancer Center of Wake Forest University

saverill@wakehealth.edu

336-713-6935

Regulatory / Budget: Megan Brown, MS

Comprehensive Cancer Center of Wake Forest University

mebrown@wakehealth.edu

336-713-6913

Investigational Drug: CPI-613

IND Number: 119491

Drug Sponsor: Cornerstone Pharmaceuticals, Inc.

Version Date: 09.09.15

11.03.14

07.17.14

03.20.14

11.12.13

09.09.13

08.13.13

**Proprietary and Confidential**

# SCHEMA

Histologic Diagnosis of Relapsed or Refractory Small Cell Lung Cancer (SCLC) Post-1st line chemotherapy

Response/Stable Disease/Clinical Benefit

Continue until clear disease progression.

Informed consent

Treatment with 2 cycles of CPI-613

Cycle length: 4 weeks

Treatment given on days 1 and 4 of weeks 1 through 3

Radiographic surveillance

Scan performed during week 4 of cycle 2, 4, 6 and every 2 cycles thereafter

No Response

Discontinue treatment

**Primary Objective**

To evaluate the response rate (RR) of CPI-613 in patients with relapsed or refractory SCLC who have failed 1st chemotherapy.

**Secondary Objective**

To evaluate the safety, toxicity, progression-free-survival (PFS) and overall survival (OS) of CPI-613 in these patients.

**Primary objective**

To evaluate the safety and anti-cancer activities (assessed based on Response Rate) of CPI-613 in patients with advanced unresectable bile duct cancer who have failed available therapies.

**Secondary Outcome measures**

- Progression-free survival
- Overall Survival

**Design**

Open-label single arm

* Maximum number of cycles allowed is 6

** Two additional cycles will not be given if the patient no longer meets eligibility criteria, has worsening of biliary function, or has symptomatic progression.

Table of Contents

[SCHEMA 3](#_Toc363723897)

[1.0 BACKGROUND AND STUDY OBJECTIVES 6](#_Toc363723898)

[1.1 Small Cell Lung Carcinoma 6](#_Toc363723899)

[1.2 CPI-613 6](#_Toc363723900)

[2.0 STUDY DESIGN 7](#_Toc363723901)

[2.1 Study Objective 7](#_Toc363723902)

[2.2 Outcome Measures 7](#_Toc363723903)

[2.3 Open-Label, Single-Arm Study Design 7](#_Toc363723904)

[3.0 PATIENT ELIGIBILITY CRITERIA 8](#_Toc363723905)

[3.1 Inclusion Criteria 8](#_Toc363723906)

[3.2 Exclusion Criteria 8](#_Toc363723907)

[3.3 Inclusion of Women and Minorities 9](#_Toc363723908)

[4.0 REGISTRATION PROCEDURES 9](#_Toc363723909)

[5.0 STUDY PROCEDURES 11](#_Toc363723910)

[5.1 Pre-Study Screening Tests and Safety Assessment 12](#_Toc363723911)

[5.1.1 Pre-Study Screening Tests 12](#_Toc363723912)

[5.1.2 Safety Assessment 12](#_Toc363723913)

[5.1.3 Tumor Assessment 12](#_Toc363723914)

[5.2 Specifics of Tests Performed During the Study 13](#_Toc363723915)

[5.2.1 ECOG Performance Status 13](#_Toc363723916)

[5.2.2 Clinical Chemistry and Hematology 14](#_Toc363723917)

[6.0 TREATMENT WITH CPI-613 14](#_Toc363723918)

[6.1 Sample Size, Dose Levels and Justification of the Dose 15](#_Toc363723919)

[6.2 Adverse Event-Related Dosing Delay and Dose Modification 15](#_Toc363723920)

[6.3 Duration of Treatment for Each Patient at Each Cohort 16](#_Toc363723921)

[7.0 STUDY DRUG - CPI-613 17](#_Toc363723922)

[7.1 Description of CPI-613 Drug Product 17](#_Toc363723923)

[7.2 Handling of CPI-613 17](#_Toc363723924)

[7.3 Storage of CPI-613 17](#_Toc363723925)

[7.4 Intravenous (IV) Infusion Sets, Syringes and IV Bags to be used for Administration of CPI-613 17](#_Toc363723926)

[7.5 Reconstitution and Administration of CPI-613 18](#_Toc363723927)

[7.6 Request for CPI-613 19](#_Toc363723928)

[7.7 Procurement of Investigational Drug 20](#_Toc363723929)

[7.8 Disposal of CPI-613 20](#_Toc363723930)

[7.9 Calculation of the Amount of CPI-613 for Each Patient 21](#_Toc363723931)

[7.10 Concomitant Medications and Prophylactic Treatment 21](#_Toc363723932)

[8.0 ADVERSE EVENTS LIST AND REPORTING REQUIREMENTS 22](#_Toc363723933)

[8.1 Adverse Event Characteristics 22](#_Toc363723934)

[8.2 STRC SAE Reporting Requirements 23](#_Toc363723935)

[8.3 WFUHS IRB AE Reporting Requirements 23](#_Toc363723936)

[9.0 STATISTICAL CONSIDERATIONS 24](#_Toc363723937)

[10.0 Data Management 25](#_Toc363723938)

[APPENDIX A – REGISTRATION GUIDELINES AND FORMS 28](#_Toc363723939)

[APPENDIX B STRC SAE REPORTING GUIDELINES 34](#_Toc363723940)

[APPENDIX C – CCCWFU ADVERSE EVENT LOG 37](#_Toc363723941)

[APPENDIX D – Data Collection Form for Efficacy Assessment 38](#_Toc363723942)

[APPENDIX E – Data collection form for telephone follow-up 39](#_Toc363723943)

[APPENDIX F – Withdrawal of participation data collection form 40](#_Toc363723944)

[APPENDIX G – Measurement Form 41](#_Toc363723945)

# 1.0 BACKGROUND AND STUDY OBJECTIVES

## 1.1 Small Cell Lung Carcinoma

SCLC accounts for ~15% of all lung cancers. SCLC is a common lung malignancy caused by tobacco smoking. There are ~30,000 cases in the US each year. Two-thirds of patients present with extensive stage disease at the time of diagnosis. In patients with extensive stage disease, chemotherapy with a traditional platinum doublet can achieve response rates of up to 80% and improve survival. However, there are few durable responses, and in the majority of these patients, disease eventually returns. Most patients relapse with relatively resistant disease and have a median survival of only 4 to 5 months, and have increased morbidity. Current guidelines from the National Comprehensive Cancer Network (NCCN) recommend clinical trials for patients with recurrent disease (http://www.nccn.org/index.asp).

## 1.2 CPI-613

CPI-613 is a novel anti-cancer agent (Zachar et al. 2011). CPI-613 selectively targets the altered form of mitochondrial energy metabolism in tumor cells, causing changes in mitochondrial enzyme activities and redox status, which leads to apoptosis, necrosis and autophagy of tumor cells (Zachar et al. 2011). The activities of CPI-613 involve the catalytic and regulatory functions of the altered form of the pyruvate dehydrogenase complex (PDC) and the α-ketoglutarate dehydrogenase complex (KGDHC) found in tumor cells (Zachar et al. 2011). Due to its novel mechanism of action and the multi-molecular targeting approach, CPI-613 is equally effective against multi-drug resistance (MDR) cancer cell lines when compared to the parental untreated cancer cell lines, according to *in vitro* studies (Zachar et al. 2011).

According to Phase I dose-escalation trials in patients with various advanced stages of solid and hematologic malignancies, CPI-613 is well-tolerated at doses up to 3,000 mg/m^2^ when infused intravenously over two hours. CPI-613 also exhibits anti-tumor activities in these patients (Pardee et al, 2011a and b, & 2012; Lee et al. 2011 & 2012; Senzer et al 2012), even in the Phase 1 patient population were those with relapsed or refractory disease.

CPI-613 was used in a 53-year old male patient (Patient DAW) with extensive stage SCLC was treated with CPI-613 and gemcitabine as a part of a Phase 1 dose-escalation trial. Patient DAW had multiple tumor lesions throughout the body including in the right lung, bone, brain, and lymph nodes in the neck, chest and pelvis. His disease had relapsed from multiple lines of chemotherapy, which included: carboplatin and etopside from 8-Jul-2009 to 19-Aug-2009 and then carboplatin alone until 14-Oct-2009 (stable disease as best response); topotecan from 9-Dec-2009 to 27-Jan-2010 (partial response as best response); and docetaxel from 3-May-2010 (disease progression). Patient DAW received whole brain radiation therapy from 3-Jun-2010 to 23-Jun-2010 (stable disease as best response). At that point, the disease was progressing rapidly and patient DAW was put on the Phase 1 trial of CPI-613. After trial registration and ~1 month of washout period per requirements of the study protocol, patient DAW began treatment with CPI-613 + gemcitabine on 10-Aug-2010. With one cycle of treatment (where a treatment cycle was CPI-613 given by IV infusion over 2 hours twice weekly for 3 weeks, concurrent with gemcitabine given once weekly for 3 weeks, followed by a week of rest), patient DAW had stable disease, despite the rapidly progressing disease immediately prior to CPI-613 treatment. Because of an adverse event unrelated to CPI-613, the patient was no longer eligible and was taken off the trial per requirements of the study protocol. The patient eventually passed away, but death occurred ~5 months later on 12-January-2011.

In summary, CPI-613 has a novel mechanism of action, is well tolerated, and exhibits anti-cancer activities against various types of solid and hematologic malignancies. This is especially noted in patients who had relapsed or refractory disease. CPI-163 does not have any signs of cross-resistance to traditional chemotherapeutic agents used in SCLC. Accordingly, the safety and anti-cancer activities of CPI-613 in patients with extensive stage SCLC who have failed two lines of chemotherapies or who have failed first line therapy and are unlikely to tolerate topotecan as second line chemotherapy are investigated in the current pilot study.

# 2.0 STUDY DESIGN

## 2.1 Study Objective

The objective of this study is to evaluate the safety and anti-cancer activities in patients with relapsed or refractory SCLC who have failed 1 or 2 lines of chemotherapy.

## 2.2 Outcome Measures

The primary outcome measure is to evaluate the response rate (RR) of CPI-613 in these patients.

The secondary outcome measures are:

- Safety
- Toxicity
- Progression-free-survival (PFS)
- Overall survival (OS)

## 2.3 Open-Label, Single-Arm Study Design

This is a single-arm open-label study, and investigators and subjects are not blinded to the treatment. Also, the assignment of patients will not be randomized, since there is only a single arm in this study.

# 3.0 PATIENT ELIGIBILITY CRITERIA

## 3.1 Inclusion Criteria

Patients must meet all of the following inclusion criteria before enrollment:

A. Histologically or cytologically proven SCLC that has relapsed or been refractory after at least one line of chemotherapy.

B. ECOG Performance Status of ≤2.

C. Must be ≥18 years of age.

D. Expected survival >1 month.

E. No acute toxicities from previous treatment higher than grade 1 at the start of treatment with CPI-613.

F. Women of child-bearing potential (i.e., women who are pre-menopausal or not surgically sterile) must use accepted contraceptive methods (abstinence, intrauterine device [IUD], oral contraceptive or double barrier device) during the study, and must have a negative serum or urine pregnancy test within 1 week prior to treatment initiation.

G. Men must practice effective contraceptive methods during the study, unless documentation of infertility exists.

H. Laboratory values ≤2 weeks prior to registration must be:

- platelet count ≥100,000 cells/mm^3^ or ≥100 bil/L
- absolute neutrophil count [ANC] ≥1500 cells/mm^3^ or ≥1.5 bil/L
- aspartate aminotransferase [AST/SGOT] ≤3x upper normal limit [UNL]
- bilirubin ≤1.5x UNL
- serum creatinine ≤1.5 mg/dL or 133 µmol/L
- albumin >3.0 g/dL or >30 g/L.

I. Mentally competent, ability to understand and willingness to sign an IRB-approved written informed consent form.

J Have access via central line (e.g., portacath) – double lumen due to CPI-613 administration requirements

## 3.2 Exclusion Criteria

Patients with the following characteristics are excluded:

A. Patients receiving any other standard or investigational treatment for their cancer, or any investigational agent for any non-cancer indication within the past 2 weeks prior to initiation of CPI-613 treatment.

B. Serious medical illness that would potentially increase patients’ risk for toxicity.

C. Any active uncontrolled bleeding or bleeding diathesis.

D. Pregnant women, women of child-bearing potential not using reliable means of contraception, or lactating women.

E. Men unwilling to practice contraceptive methods during the study period.

F. Life expectancy less than 1 month.

G. Treatment with any anti-cancer therapy within the 2 weeks prior to treatment with CPI-613.

H. Patients with untreated central nervous system (CNS) or epidural tumor.

I. Any condition or abnormality which may, in the opinion of the investigator, compromise his or her safety.

J. Unwilling or unable to follow protocol requirements.

K. Active heart disease including myocardial infarction within previous 6 months, symptomatic coronary artery disease, arrhythmias not controlled with medication, or symptomatic congestive heart failure.

N. Evidence of active infection or serious infection (e.g., septic shock with multi-organ dysfunction) within the past month.

O. Patients with known HIV infection.

P. Requirement for immediate palliative treatment of any kind including surgery.

## 3.3 Inclusion of Women and Minorities

Both men and women and members of all races and ethnic groups are eligible for participation in this trial.

# 4.0 REGISTRATION PROCEDURES

All patients entered on any CCCWFU trial, whether treatment, companion, or cancer control trial, **must** be registered with the CCCWFU Protocol Registrar or entered into the Oncology Research Information System (ORIS) Screening Log within 24 hours of informed consent.

Patients **must** be registered prior to the initiation of treatment.

In order to ensure prompt registration of your patient, please:

1. Complete the Eligibility Checklist (Appendix A)
2. Complete the Protocol Registration Form (Appendix A)
3. Alert the WFUHS registrar by phone, *and then* send the signed Informed Consent Form, Eligibility Checklist and Protocol Registration Form to the registrar, either by fax or e-mail.

Contact Information:

Protocol Registrar PHONE (336) 713-6767

Protocol Registrar FAX (336) 713-6772

Protocol Registrar E-MAIL ([registra@wakehealth.edu](mailto:registra@wakehealth.edu))

(*Protocol Registration is open from 8:30 AM - 4:00 PM, Monday-Friday.)

1. Please fax/e-mail ALL eligibility source documents with registration. Patients **will not** be registered without all required supporting documents.

**Note:** If labs were performed at an outside institution, please provide a printout of the results. Please ensure that the most recent lab values are sent.

To complete the registration process, the Registrar will:

- assign a patient study number
- register the patient on the study

# 5.0 STUDY PROCEDURES

Table 5-1 (below) provides an overview of the pre-study screen, study procedures for all treatment cycles, and the follow-up period. Specifics are described in subsequent sections.

|  | **Pre-**  **Study^**, a^** | **Every Treatment Cycle*** | | | | | | | **Follow up** |
| --- | --- | --- | --- | --- | --- | --- | --- | --- | --- |
|  |  | **Week 1** | | **Week 2** | | **Week 3** | | **Week 4** |  |
|  |  | **Day 1** | **Day 4** | **Day 1** | **Day 4** | **Day 1** | **Day 4** | **Day 3, 4 or 5** |  |
| Informed consent | X |  |  |  |  |  |  |  |  |
| Demographics | X |  |  |  |  |  |  |  |  |
| Medical history | X |  |  |  |  |  |  |  |  |
| Concurrent meds | X |  |  |  |  |  |  |  |  |
| Physical exam | X |  |  |  |  |  |  |  |  |
| Height, Weight, M^2^ | X |  |  |  |  |  |  |  |  |
| B-HCG^c^ | X |  |  |  |  |  |  |  |  |
| Current symptom evaluation^d^ | X | X |  |  |  |  |  |  |  |
| Vital signs^d^ | X | X | X | X | X | X | X |  |  |
| ECOG Status^d^ | X | X |  |  |  |  |  |  |  |
| Serum chemistry, hematology^b, d^ | X | X |  | X |  | X |  |  |  |
| CPI-613^e^ |  | X | X | X | X | X | X |  |  |
| Efficacy Assessment^f^  Contrast CT of chest / abdomen or PET/CT^f^ | X |  |  |  |  |  |  | X^f^ |  |
| Standard of care MD visit |  | X^h^ |  |  |  |  |  |  |  |
| LDH | X | X |  |  |  |  |  |  |  |
| Adverse event evaluation |  | X |  |  |  |  |  |  |  |
| Phone contact |  | | | | | | | | X |
| * Cycle Length is 4 weeks, with treatment on Days 1 and 4 of Weeks 1 through 3; week 4 is a rest  week (no treatment)  ** The only pre-study data that will be used for data analyses are ECOG status, baseline CT or PET/CT, and LDH. See section 5.1.4 for details.  a: Pre-study requirements must be completed **within** **the following time**  **frames:**  Within **4** weeks: tumor assessments based on contrast CT of chest and abdomen or PET/CT;  Within **2** weeks: medical history, physical exam, vital signs, height, weight, ECOG, evaluation of symptoms and medications, clinical chemistry, hematology, LDH  Within **1** week: pregnancy test for women of childbearing potential  b: Specific chemistries are listed in section 5.2.2. Renal function will be assessed using the Cockcroft-Gault formula.  c: Serum pregnancy test for women of childbearing potential  d: These tests are performed with results available for review within 24 hours before administration of CPI-613  e: CPI-613 is given as a 2-hr IV infusion via a central venous catheter. Treatment with CPI-613 may be adjusted +/- 1 day.  f: Imaging performed while on treatment should be preferably obtained on day 3, 4, or 5 of week 4 during cycles 2, 4,  6 and every two cycles thereafter until progression.  g: Survival and post-study cancer treatment will be monitored bimonthly via telephone contact by the study PA after  patients are taken off of the trial. Phone contact will continue indefinitely.  h: Physical exam and clinical evaluation to be completed within 72 hours prior to treatment with CPI-613 | | | | | | | | | |

## 5.1 Pre-Study Screening Tests and Safety Assessment

### 5.1.1 Pre-Study Screening Tests

Pre-study screening tests must be performed according the following time frames:

Within 4 weeks: tumor assessments based on contrast CT of chest and abdomen and/or PET/CT

Within 2 weeks: medical history, physical exam, vital signs, height, weight, ECOG, evaluation of symptoms and medications, clinical chemistry, hematology, LDH.

Within 1 week: pregnancy test for women of child-bearing potential.

### 5.1.2 Safety Assessment

The safety of CPI-613 will be assessed from the first dose to 1 month after last dose of CPI-613. The assessment will be based on:

- evaluation of symptoms

- vital signs

- ECOG performance status and survival

- clinical chemistry

- renal function

- hematology

The specifics of the safety tests are described in Section 5.2. All safety assessment tests are performed during screening (performed within 2 weeks prior to Cycle 1 treatment with CPI-613. Symptom evaluation and vital signs should be performed prior to each administration of CPI-613. ECOG performance status, clinical chemistries, renal function, and hematology should be performed on day 1 of weeks 1, 2, and 3 for each treatment cycle, with results available for review within 24 hours before administration on day 1 of study drug administration.

###

### 5.1.3 Tumor Assessment

Tumor response will be assessed based on RR, and PFS, derived from contrast CT of chest and abdomen and/or PET/CT at baseline and after every two cycles. Contrast CT and/or PET/CT should be performed during week 4, preferably on Wednesday or later (days 3, 4 or 5). RR is the primary endpoint, whereas PFS and OS are secondary endpoints.

According to the Response Evaluation Criteria in Solid Tumors (RECIST), the efficacy of treatment for solid tumors is determined by the tumor size. RECIST criteria are the golden standard to evaluate the tumor. In this study RECIST criteria will be used to evaluate response. However, there is a *pseudoprogression* phenomenon described with the use of CPI-613 in Phase I solid tumor studies (Lee et al. 2012).

It is felt that if CPI-613-induces necrosis of the tumor lesion there may be some radiographic evidence for increasing size. CPI-613 induces tumor necrosis due to its mechanism of action – selectively targeting the altered form of mitochondrial energy metabolism in tumor cells, causing changes in mitochondrial enzyme activities and redox status, which leads to apoptosis, necrosis, and autophagy of tumor cells (Lee et al., 2011; Lee et al., 2012; Senzer et al., 2012; Zachar et al., 2011; Retter et al., 2012).

For the purposes of this study, pseudoprogression will be defined and considered in patients who meet RECIST criteria of progression. It will be the treating physician’s discretion to continue treatment in this situation if the patience is deemed to be getting clinical palliative benefit. In these patients, the study drug can be given for another two cycles prior to making definitive determination of progression or response.

OS will be determined from the first dose of CPI-613 to death, assessed until patient death..Post-study survival, medical and cancer treatment information will be collected bimonthly via telephone contact after treatment termination.

RR is defined as % of patients who experienced a complete response (CR) or partial response (PR). CR and PR are based on Response Evaluation Criteria in Solid Tumor (RECIST) Version 1.1 (Eisenhauer et al. 2009). The best response recorded from the start of the treatment until disease progression (DP) will be considered.

PFS will be determined from the first dose of CPI-613 to DP or death due to any cause.

*5.1.4 Use of pre-study data for analyses*

The following pre-study tests and evaluations should be documented for future data analysis: ECOG, LDH, and CT (or PET/CT) data.

Pre-study ECOG scores and LDH values will be transferred to the Oncology Research Information System (ORIS) database at the time of registration. All subsequent ECOG scores and LDH values should be recorded on the Efficacy Assessment Form (Appendix D), which will be entered into the corresponding REDCap database. CT or PET/CT data will be recorded on the Measurement Form (Appendix G).

## 5.2 Specifics of Tests Performed During the Study

Described below are the specifics of the tests performed in this study.

### 5.2.1 ECOG Performance Status

The ECOG Performance Status scales (Oken et al 1982) will be used to assess how a patient's disease is progressing and assess how the disease affects the daily living abilities of the patient. These scales are listed in Table 5.2.1-1 (below). The higher the ECOG score, the worse the prognosis.

**Table 5.2.1-1: Scales Used in ECOG Performance Status**

| **Grade** | **ECOG** |
| --- | --- |
| 0 | Fully active, able to carry on all pre-disease performance without restriction |
| 1 | Restricted in physically strenuous activity but ambulatory and able to carry out work of a light or sedentary nature, e.g., light house work, office work |
| 2 | Ambulatory and capable of all self-care but unable to carry out any work activities. Up and about more than 50% of waking hours |
| 3 | Capable of only limited self-care, confined to bed or chair more than 50% of waking hours |
| 4 | Completely disabled. Cannot carry on any self-care. Totally confined to bed or chair |
| 5 | Dead |

### 5.2.2 Clinical Chemistry and Hematology

Clinical chemistry assessed includes:

| glucose | Ca^+2^ |
| --- | --- |
| creatinine | CO_2_ |
| total protein | BUN |
| albumin | AST/serum glutamic-oxaloacetic transaminase (SGOT) |
| Na^+^ | ALT/serum glutamic-myruvic transaminase (SGPT) |
| K^+^ | alkaline phosphatase (ALP) |
| Cl^-^ | total bilirubin |
| Mg |  |

Hematology assessed includes:

complete blood count with differential (platelet and hemoglobin included)

lactate dehydrogenase (LDH)

# 6.0 TREATMENT WITH CPI-613

CPI-613 will be administered to patients as shown in Table 6-1 (below). A treatment cycle is 4 weeks, with CPI-613 given on Days 1 and 4 of the first 3 weeks (i.e., 3-weeks-on-1-week-off). During each treatment cycle, patients will be treated with CPI-613 at the same dose, unless dose modification is warranted (see Section 6.2).

**Table 6-1: Administration of CPI-613 in Patients with Extensive Small Cell Lung Carcinoma**

| **Treatment Cycle** | | | **Administration of CPI-613** |
| --- | --- | --- | --- |
|  | **Week** | **Day** |  |
| **Cycle 1**  **(4 weeks)** | 1 | 1 | 2-hr IV infusion via a central venous catheter |
|  |  | 4 | 2-hr IV infusion via a central venous catheter |
|  | 2 | 1 | 2-hr IV infusion via a central venous catheter |
|  |  | 4 | 2-hr IV infusion via a central venous catheter |
|  | 3 | 1 | 2-hr IV infusion via a central venous catheter |
|  |  | 4 | 2-hr IV infusion via a central venous catheter |
|  | 4 |  |  |
| **Cycle 2**  **(4 weeks)** | 1 | 1 | 2-hr IV infusion via a central venous catheter |
|  |  | 4 | 2-hr IV infusion via a central venous catheter |
|  | 2 | 1 | 2-hr IV infusion via a central venous catheter |
|  |  | 4 | 2-hr IV infusion via a central venous catheter |
|  | 3 | 1 | 2-hr IV infusion via a central venous catheter |
|  |  | 4 | 2-hr IV infusion via a central venous catheter |
|  | 4 |  |  |
| Etc. until progression |  |  |  |

hr = hour; IV = intravenous; min = minutes.

## 6.1 Sample Size, Dose Levels and Justification of the Dose

The sample size will be 20 for this pilot trial.

This study will use the maximum tolerated dose (MTD) established in the study conducted by Pardee and colleagues (CCCWFU 29109; IRB00012124). This MTD for single-agent use of CPI-613 infused over 2 hours is 3,000 mg/m^2^.

## 6.2 Adverse Event-Related Dosing Delay and Dose Modification

For adverse events unrelated to serum creatinine elevation or reduction in renal function but are possibly related to CPI-613, the occurrence of Grade 1 toxicity does not generally require dose modification for subsequent doses for that patient. However, if Grade 2 toxicity (other than alopecia and nausea) probably related to CPI-613 develops, treatment is to be withheld and can resume only after the Grade 2 toxicity has been reduced to Grade 1 or below, and the dose level for subsequent doses for that patient will be reduced by 25% of the dose at which such Grade 2 toxicity occurs. Grade 2 alopecia and nausea do not require withholding treatment or dose reduction. If Grade 3 or 4 toxicity probably related to CPI-613 develops, dosing of CPI-613 of that patient will be withheld and the patient shall be monitored for recovery from, and reversibility of, such Grade 3 or 4 toxicity. To resume treatment with CPI-613 for a patient who has had CPI-613-related Grade 3 or 4 toxicity, the Grade 3 or 4 toxicity must be reduced to Grade 1 or below, and the dose level for subsequent doses for that patient will be reduced to 50% of the dose at which such Grade 3 or 4 toxicity occurs.

For adverse events related to creatinine elevation or reduction in renal function that are possibly related to CPI-613, dosing of the patient will be withheld if the severity level is Grade 1 or above. Treatment can resume only after the toxicity has been reduced to Grade 0. The dose level for subsequent doses for that patient will be reduced by 15% if the severity level is of Grade 1, by 25% for Grade 2 toxicity, and by 50% for Grade 3 or 4 toxicity.

Furthermore, if the toxicity possibly related to CPI-613 is acute renal failure and the severity level is Grade 3 or 4, further patient enrollment will be temporarily suspended in order to enable assessment of the following aspects of the trial and implementation of corrective measures or protocol amendment, and if necessary:

- compliance of the study sites and investigators to the study protocol

- evaluation of the appropriateness of the procedures for monitoring renal function

## 6.3 Duration of Treatment for Each Patient at Each Cohort

Six months of treatment is recommended for patients who have a response, unless or until:

- Patients exhibit progression of disease together with worsening of biliary function, no longer meeting eligibility criteria, or exhibiting symptomatic progression; or patients exhibit progression of disease which was confirmed after 2 additional treatment cycles

- Unacceptable toxicity from CPI-613

- Patient withdrawal of consent

- Investigator’s discretion to withdraw patients from the study because continued participation in the study is not in the patient’s best interest.

- Undercurrent illness: a condition, injury, or disease unrelated to the intended disease for which the study is investigating, that renders continuing the treatment unsafe or regular follow-up impossible

- General or specific changes in the patient's condition that renders the patient ineligible for further investigational treatment

- Non-compliance with investigational treatment, protocol-required evaluations or follow-up visits

- Termination of the clinical trial by the sponsor

However, if therapy is considered beneficial according to the opinion of the treating physician, additional cycles of therapy may be given on the same treatment plan indefinitely up to the time of clear disease progression.

When terminating treatment during this trial, the investigator should make every effort to contact the patient and to perform a final evaluation. Also, the reason(s) for withdrawal from the study must be recorded.

Survival and post-study cancer treatment will be monitored via bi-monthly telephone contact once patients are removed from trial. All patients will be followed until death.

# 7.0 STUDY DRUG - CPI-613

## 7.1 Description of CPI-613 Drug Product

CPI-613 is provided in 10-mL amber glass vials. Each vial contains 10 mL of CPI-613 at a concentration 50 mg/mL, equivalent to 500 mg of CPI-613. The drug product of CPI-613 is a clear and colorless solution that is free of any particulate matter.

## 7.2 Handling of CPI-613

CPI-613 is an investigational drug and its toxicity in humans is not fully understood. All necessary precautions in handling potentially toxic chemicals must be strictly adhered to. Gloves and protective clothing must be worn when handling CPI-613. Avoid contact by all modes of exposure. If the solution contacts the skin, it must be washed immediately and thoroughly with soap and water. If the solution comes in contact with mucous membranes, the membranes must be flushed thoroughly with water. Spills should be picked up with absorbent material and the area must be washed at least 3 times with ethyl alcohol followed by water.

CPI-613 drug product is slightly photosensitive (Study# PHO-001). Therefore, after removal of CPI-613 drug product from the amber vials, CPI-613 drug product should be protected from excessive light before administration to patients.

## 7.3 Storage of CPI-613

CPI-613 can be stored at room temperature and preferably stored under refrigeration, at 2˚-8˚C (36˚-46˚F), except when being prepared for administration.

## 7.4 Intravenous (IV) Infusion Sets, Syringes and IV Bags to be used for Administration of CPI-613

CPI-613 must be administered IV by infusion, via an IV catheter with D5W running at a rate of about 125-150 mL/hr. To avoid local reactions at and around the site of administration, CPI-613 should be administered via a central venous catheter. Subsequent sections describe the appropriate types of IV catheters, IV bags, syringes and clinical solutions that can be used in mixing and administering CPI-613 to patients.

Leaching of Diethylhexyl Phthalate (DEHP): CPI-613 can cause leaching of DEHP from IV infusion sets and IV bags (Study COM-003). Therefore, DEHP-containing IV infusion sets, IV bags or syringes should not be used in mixing or administration of CPI-613. Examples of the IV sets, IV bags and syringes that do not contains DEHP and therefore can be used in the administration of CPI-613 are:

**Extension Set for Syringe Pump Use: All extension sets from MED-RX do not contain DEHP.**

Syringes: A**ll Monoject syringes are DEHP free.**

IV Infusion Sets: A compatibility study has been conducted showing that CPI-613 is compatible with 4 commonly used IV infusion sets (Study# COM-001). Therefore, these 4 types of IV infusion sets, and IV infusion sets that are made with the same materials, can be used to administer CPI-613. These IV infusion sets are:

- PVC material - ADDitIV^®^ Primary IV Set with Universal Spike, Backcheck Valve, 2 Injection Sites, DEHP-Free and Latex-Free, 15 drops/mL, REF V14453, B Braun Medical Inc.
- Latex material - Interlink^®^ System Secondary Medication Set, 10 drops/mL, 2C7451, Baxter Healthcare Corporation
- PVC material - Surshield^TM^ Safety Winged Infusion Set, 0.19 mL Volume, Latex-Free, DEHP-Free, SV*S25BLS, Terumo Medical Products Hangzhou Co. Ltd.
- Polyethylene material - Interlink^®^ System Paclitaxel Set by Baxter HealthCare, Non DEHP-free:  Polyethylene tubing with a 0.22 microfilter Item # 2C7558 10 drops/mL

Syringes: Compatibility studies (Studies# COM-001 and COM-002) have shown that CPI-613 drug product (50 mg/mL), and drug product diluted with D5W to various concentrations (1.6-25 mg/mL) are compatible with various types of syringes, as listed below. Therefore, any of these types of syringes, and syringes that are made with the same materials, can be used to administer CPI-613. Also, glass syringes can also be used, since glass (such as glass containers) is compatible with CPI-613 drug product.

- Norm-Ject, polyethlyene barrel, polyethylene plunger, latex free (Henke Sass Wolf GMBH) syringes
- Becton Dickinson syringes
- Terumo syringes
- Monoject syringes
- Glass syringes

## 7.5 Reconstitution and Administration of CPI-613

CPI-613 must be diluted from 50 mg/mL to 12.5 mg/mL with 5% Dextrose Water or D5W (i.e., 1 portion of CPI-613 diluted with 3 portions of D5W) prior to administration. The diluted drug product should be visually inspected for clarity. If haziness, precipitate or coloration (other than colorless) is observed, do not use the diluted drug product for dosing. After dilution with sterile D5W, the solution is clear and has a pH of 8.4-8.8. The diluted CPI-613 drug product has been found to be stable for 24 hrs at room temperature and refrigeration temperature (Studies STA-010).

CPI-613 must be administered IV, via an IV catheter that is free flowing and free of air in the dead space of the IV catheter, to minimize vascular irritation, inflammation and acute toxicity of CPI-613 (Study NCL-049). Accidental co-administration of extra air in the dead space of IV catheters during administration of CPI-613 has demonstrated the potential to induce acute toxicity of CPI-613 according to animal studies (Study NCL-049). Also, accidental leakage of CPI-613 into the perivascular space during IV administration, which prolongs exposure of perivascular tissue to CPI-613, can induce significant local inflammation according to animal studies (Studies NCL-027 and NCL-030). To avoid local reactions at and around the site of administration, CPI-613 must be administered via a central venous catheter.

CPI-613 must not be administered as a bolus, but by infusion, at a rate of ~0.5 mL/min, via a central venous catheter with D5W running at a rate of about 125-150 mL/hr. This is to minimize potential acute toxicity of CPI-613, according to animal studies (Study NCL-049) and requires a double lumen Port a Cath.

The following precautions must be taken when administering CPI-613:

A. Confirmation of the placement of the IV line to ensure a lack of leakage of CPI-613 into the perivascular space.

B. Confirmation that the IV line is free flowing.

C. Confirmation that the IV line is free of dead air space.

D. Dilute CPI-613 drug product with D5W, as instructed in the study protocol.

E. Administer CPI-613 by infusion, not as a bolus.

F. After administration of CPI-613, flush the IV line with ~10 mL of D5W to remove residual CPI-613.

G. To avoid local reactions at and around the site of administration, CPI-613 should be administered via a double lumen central venous catheter.

## 7.6 Request for CPI-613

CPI-613 must be requested from Cornerstone by the Principal Investigator (or authorized designees). CPI-613 may not be used outside the scope of this protocol, nor can it be transferred or licensed to any party not participating in this clinical study. Cornerstone policy requires that CPI-613 be shipped directly to the institution where the patient is to be treated. Cornerstone does not permit the transfer of CPI-613 between institutions (unless with prior written approval from Cornerstone). Requests must be submitted to Cornerstone by fax or email to the following address:

Ms. Claudia Maturo

Department of Regulatory and Clinical Affairs

Cornerstone Pharmaceuticals, Inc.

25 Health Sciences Drive

Stony Brook, NY 11790

Telephone: 631-444-6868

Telefax: 631-444-6895

Email: claudia@cornerstonepharma.com

The following information must be provided in the request of CPI-613 from Cornerstone:

- Names of the principal investigator and the requestor (if different)

- Name of the study site

- Name of the pharmacist responsible for receiving and storing CPI-613

- Name of the person and address where CPI-613 is to be shipped to

- Amount (# vials) requested

- Date of request

- Date shipment expected

- Study Protocol (title and protocol#) for which the requested CPI-613 is to be used

## 7.7 Procurement of Investigational Drug

Relevant regulations require investigators to establish a record of the receipt, use and disposition of all investigational products. Investigators may delegate responsibility of drug ordering, storage, accountability and preparation to their designees.

The investigator, or the designee, will be responsible for dispensing and accounting of CPI-613 provided by Cornerstone and for exercising accepted medical and pharmacy practices.

Records of inventory, dispensation and disposition (vials received, source and dates) must be maintained. In addition, all doses dispensed should be accounted for by recording the date, study number and name, patient identification, patient initials, patient medical record number and balance forward. These records must be maintained and kept at the study site, and will be reviewed by Cornerstone, or its designee, during periodic monitoring visits.

## 7.8 Disposal of CPI-613

The following procedures are to be taken in disposal of CPI-613:

- During the study, store the used CPI-613 vials (which must be separate from the unused CPI-613 vials) at room temperature in an access-limited area. Alternatively, destroy the used CPI-613 vials according to institutional policy after documentation of the number of used CPI-613 vials and remaining volume in each used vial.

- At the end of the study, deface the label (both used and unused vials) with a permanent marking pen.

- For used CPI-613 vials (if not already destroyed according to institutional policy), after documentation of the number of used CPI-613 units and remaining volume in each container, the used containers should be destroyed at the site according to the institutional procedures for destroying toxic chemicals. A certificate documenting the destruction of used vials must be kept on file.

- All unused CPI-613 vials must be destroyed according to the policy of the institution. The destruction of CPI-613, and the quantity destroyed, must be documented. A copy of the Certificate of Destruction should be sent to:

Ms. Claudia Maturo

Department of Regulatory and Clinical Affairs

Cornerstone Pharmaceuticals, Inc.

25 Health Sciences Drive

Stony Brook, NY 11790

Telephone: 631-444-6868

Telefax: 631-444-6895

Email: claudia@cornerstonepharma.com

## 7.9 Calculation of the Amount of CPI-613 for Each Patient

The amount of CPI-613 at each dose level is based on the BSA of the patient. The BSA values will be calculated based on the height and body weight taken during screening and this BSA value is used throughout the study. This is unless there is a >10% change in the body weight from baseline during the study. At that point, BSA should be revised based on the new body weight and height. The new BSA values will be used from that point on for the remainder of the study, unless there is another >10% change in body weight which will require another revision of the BSA.

## 7.10 Concomitant Medications and Prophylactic Treatment

Patients cannot receive any standard or investigational treatment (except CPI-613) for their cancer, or any other investigational drugs for any non-cancer indications, while on this study. All otherwise permitted concomitant medications (including trade and generic names, dosage and dosing schedule) must be recorded. Treatment of disease-related symptoms (such as nausea) is permitted. Medications administered in such instances will be considered concomitant medications and should be documented accordingly.

Prophylactic treatment for drug-related symptoms is not planned, or it will interfere with the assessment of the toxicity of the investigational product. However, following the evaluation of the causal relationship of the symptom(s) to the study drug and the information has been documented, the investigator may prescribe supportive treatment. Supportive treatment may include anti‑emetic, anti‑diarrhea, anti‑pyretic, anti‑allergic, anti‑hypertensive medications, analgesics, antibiotics, allopurinol, and others such as blood products and bone marrow growth factors. Patients may use erythropoietin for chronic anemia. Also, the hemoglobin should be maintained ≥8 g/dL during the course of the study. The treating physician may utilize erythropoietic factors, or blood or platelet transfusions at their discretion.

# 8.0 ADVERSE EVENTS LIST AND REPORTING REQUIREMENTS

## 8.1 Adverse Event Characteristics

- **CTCAE term (AE description) and grade:** The CTEP Active Version of the NCI Common Terminology Criteria for Adverse Events (CTCAE 4.0) will be utilized for AE reporting.  The CTEP Active Version of the CTCAE is identified and located on the CTEP website at (<http://ctep.cancer.gov/protocolDevelopment/electronic_applications/ctc.htm>.  All appropriate treatment areas should have access to a copy of the CTEP Active Version of CTCAE.
- **‘Expectedness’**: AEs can be ‘Unexpected’ or ‘Expected’ (see Section 7.1 above) for expedited reporting purposes only.
- **Attribution** of the AE:
  - Definite – The AE *is clearly related* to the study treatment.
  - Probable – The AE *is likely related* to the study treatment.
  - Possible – The AE *may be related* to the study treatment.
  - Unlikely – The AE *is doubtfully related* to the study treatment.
  - Unrelated – The AE *is clearly NOT related* to the study treatment.

**List of Adverse Events to be Reported:**

*Abdominal pain

*Alkaline phosphatase

*ALT (SGPT)

*Anorexia

*AST (SGOT)

*Bilirubin (hyperbilirubinemia)

*Calcium (hypercalcemia, hypocalcemia)

*Creatinine

*Diarrhea

*Flushing

*Hemoglobin (anemia)

*Injection site Reaction

*Leukocytes

*Lymphopenia

*Nausea

*Neutrophils (neutropenia)

*Platelets (thrombocytopenia)

*Potassium

*Sodium

*Vomiting

All adverse events should be reported in the AE reporting log (Appendix C) and in ORIS regardless of whether they are on this list. Asterisk (*) denotes expected Adverse Events.

All Serious Adverse Events (SAEs) which are Possibly, Probably or Definitively Related to CPI-613, and Unexpected are required to be reported to Cornerstone Pharmaceuticals via the provided SAE Reporting Form within 7 days. All completed forms must be sent to Claudia Moore at Cornerstone. Cornerstone will submit any applicable SAEs to the FDA.

SAE reports must be submitted to Cornerstone by fax or email to the following address:

Department of Regulatory and Clinical Affairs

Cornerstone Pharmaceuticals, Inc.

25 Health Sciences Drive

Stony Brook, NY 11790

Telephone: 631-444-6868

Telefax: 631-444-6895

Email: [claudiamoore2001@yahoo.com](mailto:claudiamoore2001@yahoo.com)

## 8.2 STRC SAE Reporting Requirements

The Safety and Toxicity Review Committee (STRC) is responsible for reviewing SAEs for CCCWFU Institutional studies as outlined in Appendix B. STRC currently requires that all unexpected grade 4 and all grade 5 SAEs on these trials be reported to them for review. This procedure is a part of the CCCWFU Data Safety Monitoring Plan that our institution has on file at the NCI. All CRM staff members assisting a PI in investigating, documenting and reporting an SAE qualifying for STRC reporting are responsible for informing a clinical member of the STRC committee as well as the entire committee via the email notification procedure of the occurrence of an SAE.

## 8.3 WFUHS IRB AE Reporting Requirements

Any unanticipated problems involving risks to subjects or others and adverse events shall be promptly reported to the IRB, according to institutional policy. Reporting to the IRB is required regardless of the funding source, study sponsor, or whether the event involves an investigational or marketed drug, biologic or device. Reportable events are not limited to physical injury, but include psychological, economic and social harm. Reportable events may arise as a result of drugs, biological agents, devices, procedures or other interventions, or as a result of questionnaires, surveys, observations or other interactions with research subjects.

All members of the research team are responsible for the appropriate reporting to the IRB and other applicable parties of unanticipated problems involving risk to subjects or others. The Principal Investigator, however, is ultimately responsible for ensuring the prompt reporting of unanticipated problems involving risk to subjects or others to the IRB. The Principal Investigator is also responsible for ensuring that all reported unanticipated risks to subjects and others which they receive are reviewed to determine whether the report represents a change in the risks and/or benefits to study participants, and whether any changes in the informed consent, protocol or other study-related documents are required.

Any unanticipated problems involving risks to subjects or others occurring at a site where the study has been approved by the WFUHS IRB (internal events) must be reported to the WFUHS IRB within 7 calendar days of the investigator or other members of the study team becoming aware of the event.

Any unanticipated problems involving risks to subjects or others occurring at another site conducting the same study that has been approved by the WFUHS IRB (external events) must be reported to the WFUHS IRB within 7 calendar days of the investigator or other members of the study team becoming aware of the event.

Any event, incident, experience, or outcome that alters the risk versus potential benefit of the research and as a result warrants a substantive change in the research protocol or informed consent process/document in order to insure the safety, rights or welfare of research subjects.

# 9.0 STATISTICAL CONSIDERATIONS

This is a pilot study with the primary goal to be able to determine initial estimates of response rate (RR), and secondary goals to determine progression-free survival (PFS) and overall survival (OS). Data from this trial will also provide additional data concerning the safety profile of the CPI-613 therapy. We now describe anticipated accrual rates, proposed analyses, and sample size considerations.

Accrual Rate

We anticipate accruing one to two patients per month for this protocol. Thus, we anticipate that we will reach the target sample size of 20 patients treated at MTD within eighteen months.

Proposed Analyses

The analyses for this protocol will be primarily descriptive. With a sample size of 20 treated at MTD, we will be able to get initial estimates of RR, PFS, OS, and safety and toxicity.

For the primary outcome of tumor response rate we will present the proportion of patients who are CR, PR, SD or PD (Complete response, Partial response, stable disease and progressive disease, respectively). In addition, we will estimate the proportion of responders as the percent of patients who are SD, PR and CR, as well as RR (sum of PR + CR) and Disease Control Rate (DCR, which is the sum of SD, PR and CR). For these rates we will also include 95% confidence intervals.

For the time-to-event outcomes, we will estimate survival curves for OS and PFS using Kaplan-Meier techniques. In addition, we will estimate the 6 month and 1-year OS and PFS rates for these participants. We will estimate median overall and progression free survivals for these patients as well. We will also examine safety and toxicities for these 20 patients by looking at each toxicity identified earlier in the protocol by grade.

Sample Size Considerations

Since this is a single arm pilot study, our primary goal is to gather preliminary data on response rates and not to test a specific hypothesis per se. With this sample size we will be able to estimate the response rate using a 2-sided 95% confidence interval with an interval that will extend no more than 0.21 from the observed response rate (if we conservatively assume a response rate of 0.5 for calculations).

# 10.0 Data Management

| Informed consent document | At time of registration | ORIS |
| --- | --- | --- |
| Protocol registration form | At time of registration | ORIS |
| Efficacy assessment form (Appendix D) | Within 1 week | REDCap |
| Phone follow-up form (Appendix E) | Within 1 week | REDCap |
| Withdrawal of participation form (Appendix F) | Within 1 week | REDCap |
| Tumor measurement form (Appendix G) | Within 1 week | REDCap |

10.0 REFERENCES

1. Eisenhauer EA, Therasse P, et al. 2009. New response evaluation criteria in solid tumours: Revised RECIST guideline (version 1.1). European Journal of Cancer. **45**:228-247.
2. Lee K, Khaira D, Rodriguez R, Maturo C, O’Donnell K, Shorr R. Long-Term Stable Disease of Stage IV Pancreatic Neuroendocrine Tumors and Without Significant Adverse Effect by CPI-613, an Investigational Novel Anti-Cancer Agent. Case Study and Case Report, 1(3):137-145, 2011.
3. Lee K, Maturo C, Luddy J, Rodriguez R, Shorr R. Pseudo-progression of metastatic pancreatic cancer assessed by imaging studies - a case report. Case Study and Case Report 2(3): 95-101,2012.
4. Oken MM, Creech RH, Tormey DC, Horton J, Davis TE, McFadden ET, Carbone PP. Toxicity And Response Criteria Of The Eastern Cooperative Oncology Group. Am J Clin Oncol 5:649-655, 1982.
5. Pardee TS, DeFord-Watts LM, Peronto E, Levitan DA, Hurd DD, Kridel S. Altered lipid and mitochondrial metabolism are viable targets in acute leukemia. American Society of Hematology (ASH). Abstract# 3618. 53^rd^ Annual Meeting and Exposition, 2011a.
6. Pardee TS, Levitan DA, Hurd DD. Altered mitochondrial metabolism as a target in acute myeloid leukemia. J Clin Oncol 29: 2011b (suppl; abstr 6590)
7. Pardee TS, DeFord-Watts LM, Peronto E, Levitan DA, Hurd DD, Kridel S, Harrelson R, Manuel M, Lyerly S, Powell BL. Evaluation of the first-in-class antimitochondrial metabolism agent CPI-613 in hematologic malignancies. J Clin Oncol 30: 2012 (suppl; abstr 6524)
8. Senzer N, Bedell C, Maturo C, Luddy J, Shorr R, Lee K. CPI-613, an investigational novel anti-cancer agent, provides long-term stable disease without significant adverse effects in a patient with stage IV relapsed hepatocellular carcinoma. Case Study and Case report 2012; 2(2): 38-45,2012.
9. Study COM-001. 2006. Compatibility Testing of CPI-613 Drug Product - Infusion Sets.
10. Study COM-002. Bhasin R. 2008. Compatibility Testing of CPI-613 Drug Product Dosing Solutions with Different Types of Syringes.
11. Study COM-003. Gupta D. 2009. Investigation of the Release Behavior of Diethylhexyl Phthalate (DEHP) from the Polyvinyl-Chloride Containing Infusion Sets and Bags for Intravenous Administration by CPI-613 Drug Product.
12. Study NCL-027. Seng J. 2006. Escalating Dose Toxicology Study of Intravenously Administered CPI-613 to Miniature Pigs.
13. Study NCL-030. Seng J. 2006. An Acute Toxicity Study of CPI-613 Administered Via the Intravenous (Slow Bolus) Route to Mice.
14. Study NCL-049. Moore C, Karnik S, Lee K. 2007. Preliminary Studies of Comparative Toxicity of CPI-613 Administered Intravenously (IV) as Bolus Vs. Infusion, Effects of Air in Dead Space of Butterfly IV Infusion Set on Toxicity of CPI-613, and Acute Effects of CPI-613 on Clinical Chemistry in Rats.
15. Study PHO-001 (Covance Study# 7769-101). Potts B. 2007. Photostability Testing of CPI-613 Drug Product.
16. Study STA-010. Bhasin R. 2007. Stability of CPI-613 Injection dosing solutions after Dilution with 5% Dextrose (D5W).
17. Study VLD-002 (CR Study# LRH00016LX). McFarlene J. 2006. Validation of a High Performance Liquid Chromatographic-Mass Spectrometric Method for the Analysis of CPI-613 in K3 and K2 EDTA Human Plasma.
18. Zachar Z, Marecek J, Maturo Claudia, Gupta S, Stuart S, Howell K, Schauble A, Lem J, Piramzadian A, Karnik S, Lee K, Rodriguez R, Shorr R, Bingham PM: Non-redox-active lipoate derivates disrupt cancer cell mitochondrial metabolism and are potent anti-cancer agents *in vivo*. J Mol Med 19 July 2011 Online. DOI 10.1007/s00109-011-0785-8.

# APPENDIX A – REGISTRATION GUIDELINES AND FORMS

The following guidelines have been developed in order to ensure timely registration of your patient.

All patients entered on any CCCWFU trial, whether treatment, companion, or cancer control trial, **must** be registered with the CCCWFU Protocol Registrar or entered into ORIS Screening Log within 24 hours of Informed Consent. Patients **must** be registered prior to the initiation of treatment.

In order to ensure prompt registration of your patient, please:

1. Complete the Eligibility Checklist (attached)
2. Complete the Protocol Registration Form (attached)
3. Alert the WFUHS registrar by phone, *and then* send the signed Informed Consent Form, Eligibility Checklist and Protocol Registration Form to the registrar, either by fax or

e-mail.

Contact Information:

Protocol Registrar PHONE (336) 713-6767

Protocol Registrar FAX (336) 713-6772

Protocol Registrar E-MAIL ([registra@wakehealth.edu](mailto:registra@wfubmc.edu))

*Protocol Registration is open from 8:30 AM - 4:00 PM, Monday-Friday.

1. Please fax/e-mail ALL eligibility source documents with registration. Patients **will not** be registered without all required supporting documents.

**Note:** If labs were performed at an outside institution, please provide a printout of the results. Please ensure that the most recent lab values are sent.

| **CCCWFU # Eligibility Checklist** | | | | |
| --- | --- | --- | --- | --- |
| **Yes** | **No** | **N/A** | Inclusion Criteria (All responses must be **YES** in order to enter study) | **Eligibility Confirmation (registrar)** |
| 🞎 | 🞎 | 🞎 | 1. Does the patient have histologically or cytologically proven extensive stage SCLC that has relapsed or been refractory after at least one line of chemotherapy. |  |
| 🞎 | 🞎 | 🞎 | 1. Does the patient have an ECOG performance status of < 2? |  |
| 🞎 | 🞎 | 🞎 | 1. Is the patient 18 years of age or older? |  |
| 🞎 | 🞎 | 🞎 | 1. Does the patient have an estimated survival of > 1 month? |  |
| 🞎 | 🞎 | 🞎 | 1. Is the patient free from acute toxic effects from previous treatment superior to grade 1 at the start of treatment with CPI-613? |  |
| 🞎 | 🞎 | 🞎 | 1. If the patient is a woman of child-bearing potential (i.e., pre-menopausal or not surgically sterile), does she agree to use accepted contraceptive methods (abstinence, intrauterine device [IUD], oral contraceptive or double barrier device) during the study, and agree to have a negative serum or urine pregnancy test within 1 week prior to treatment initiation? |  |
| 🞎 | 🞎 | 🞎 | 1. If the patient is a fertile man, does he agree to practice effective contraceptive methods during the study, unless documentation of infertility exists? |  |
| 🞎 | 🞎 | 🞎 | 1. Does the patient have documentation of the following laboratory values?  - platelet count ≥100,000 cells/mm^3^ or ≥100 bil/L - absolute neutrophil count [ANC] ≥1,500 cells/mm^3^ or ≥1.5 bil/L - aspartate aminotransferase [AST/SGOT] ≤3x upper normal limit [UNL] - bilirubin ≤1.5x UNL - serum creatinine ≤1.5 mg/dL or 133 µmol/L - Albumin >3.0 g/dL or >30 g/L. |  |
| 🞎 | 🞎 | 🞎 | 1. Is the patient mentally competent, able to understand and willing to sign an IRB-approved written informed consent document? |  |
| 🞎 | 🞎 | 🞎 | 1. Does the patient have access via a double lumen central line (e.g., portacath)? |  |
|  | | | | |
| **Yes** | **No** | **N/A** | Exclusion Criteria (All responses must be **NO** in order to enter study) | **Eligibility Confirmation (registrar)** |
| 🞎 | 🞎 | 🞎 | 1. Is the patient receiving any other standard or investigational treatment for their cancer, or have they received any other investigational agent for any indication within the past 2 weeks prior to initiation of CPI-613 treatment? |  |
| 🞎 | 🞎 | 🞎 | 1. Does the patient have a serious medical illness that would potentially increase his or her risk for toxicity? |  |
|  |  |  | 1. Does the patient have any active uncontrolled bleeding or bleeding diathesis? |  |
| 🞎 | 🞎 | 🞎 | 1. Is the patient a pregnant woman, or a woman of child-bearing potential who is not using reliable means of contraception? |  |
| 🞎 | 🞎 | 🞎 | 1. Is the patient a lactating female? |  |
| 🞎 | 🞎 | 🞎 | 1. Is the patient a fertile man who is unwilling to practice contraceptive methods during the study period? |  |
| 🞎 | 🞎 | 🞎 | 1. Is the patient’s life expectancy less than 1 month? |  |
| 🞎 | 🞎 | 🞎 | 1. Has the patient received treatment with any anti-cancer therapy within the 2 weeks prior to treatment with CPI-613? |  |
| 🞎 | 🞎 | 🞎 | 1. Does the patient have an unrelated central nervous system (CNS) or epidural tumor? |  |
| 🞎 | 🞎 | 🞎 | 1. Does the patient have any condition or abnormality which may, in the opinion of the investigator, compromise his or her safety? |  |
| 🞎 | 🞎 | 🞎 | 1. Is the patient unwilling or unable to follow protocol requirements? |  |
| 🞎 | 🞎 | 🞎 | 1. Does the patient have active heart disease including myocardial infarction within previous 6 months, symptomatic coronary artery disease, arrhythmias not controlled with medication, or symptomatic congestive heart failure? |  |
| 🞎 | 🞎 | 🞎 | 1. Does the patient have evidence of active infection, or serious infection (e.g septic shock with multi-organ dysfunction) within the past month? |  |
| 🞎 | 🞎 | 🞎 | 1. Does the patient have known HIV infection? |  |
| 🞎 | 🞎 | 🞎 | 1. Does the patient require immediate palliative treatment of any kind, including surgery? |  |

Signature: ___________________________________ Date: _________________

*Please send source documentation with Eligibility Form.*

**CCCWFU # 62113 Protocol Registration Form**

| **DEMOGRAPHICS** | | | | |
| --- | --- | --- | --- | --- |
| Patient: Last Name: _____________________ | | | First Name: ____________________________ | |
| MRN: ___ ___ ___ ___ ___ ___ ___ ___ | | | DOB (mm/dd/yy): ___ ___ / ___ ___ / ___ ___ | |
| SEX: | 🞎 Male  🞎 Female | | Ethnicity (choose one): | 🞎 Hispanic  🞎Non-Hispanic |
| Race (choose all that apply): | | 🞎 WHITE 🞎BLACK 🞎 ASIAN  🞎 PAIFIC ISLANDER 🞎 NATIVE AMERICAN | | |
| Height: ___ ___**.**___ inches | | | Weight: ___ ___ ___**.**___lbs.(actual) | |
| Surface Area: ___ ___**.**___m^2^ | | |  | |
| Zip Code: ___ ___ ___ ___ ___  Primary Diagnosis: _____________________________________________________________ | | | | |
| Date of Diagnosis: ___ ___ /___ ___/___ ___ | | |  | |

ECOG performance status: __________________________________

Prior Treatments for Lung Cancer:

Name Duration

________________________________ ___________________________________

________________________________ ___________________________________

________________________________ ___________________________________

________________________________ ___________________________________

________________________________ ___________________________________

________________________________ ___________________________________

| **PROTOCOL INFORMATION** | |
| --- | --- |
| Date of Registration: | ___ ___ / ___ ___ / ___ ___ |
| MD Name (last) : | ______________________ |
| Date protocol treatment started: | ___ ___ / ___ ___ / ___ ___ |
| Informed written consent:  (consent must be signed prior to registration) | 🞎 YES 🞎 NO |
| Date Consent Signed: | ___ ___ / ___ ___ / ___ ___ |
| PID # (to be assigned by ORIS): | ___ ___ ___ ___ ___ ___ ___ ___ |

*Protocol Registrar can be contact by calling 336-713-6767 between 8:30 AM and 4:00 PM, Monday – Friday.*

*Completed Eligibility Checklist and Protocol Registration Form must be hand delivered, faxed or e-mailed to the registrar at 336-7136772 or* [*registra@wakehealth.edu*](mailto:registra@wakehealth.edu)*.*

**CCCWFU # 62113 Eligibility Source Documentation Checklist**

**(to be submitted with Protocol Registration Form)**

| **Source Documents Needed (see eligibility form above for changes)** | | | **✓ or N/A** | |
| --- | --- | --- | --- | --- |
|  | Pathology report confirming histologically or cytologically proven extensive stage SCLC that has relapsed or been refractory after one line of chemotherapy? |  | |  |
|  | Lab report documenting all initial required lab values |  | |  |
|  | Documentation that the patient is free from acute toxic effects from previous treatment superior to grade 1 |  | |  |
|  | Documentation of ECOG performance status of 0, 1, or 2 |  | |  |
|  | Documentation of expected survival > 1 month |  | |  |
|  | Most recent H&P documenting patient is 18 years of age or older |  | |  |
|  | For female patients of child-bearing potential (i.e., pre-menopausal or not surgically sterile), documentation that the patient agrees to use accepted contraceptive methods (abstinence, intrauterine device [IUD], oral contraceptive or double barrier device) during the study, and agrees to have a negative serum or urine pregnancy test within 1 week prior to treatment initiation. |  | |  |
|  | For male patients, documentation of infertility OR for fertile men, documentation that patient agrees to practice effective contraceptive methods during the study |  | |  |
|  | Lab report performed (< 2 weeks) documenting the following values:   - Platelet count ≥100,000 cells/mm^3^ or ≥100 bil/L - Absolute neutrophil count ≥1500 cells/mm^3^ or ≥1.5 bil/L - Aspartate aminotransferase [AST/SGOT] ≤3x upper normal limit [UNL] - Bilirubin ≤1.5x UNL - Serum creatinine ≤1.5 mg/dL or 133 µmol/L - Albumin > 3.0g/dL or 30 g/L |  | |  |
|  | Documentation that the patient is mentally competent, able to understand and willing to sign an IRB-approved written informed consent document |  | |  |
|  | Copy of signed consent form |  | |  |
|  | Documentation that the patient is not receiving any other standard or investigational treatment for their cancer, or has received any other investigational agent for any indication within the past 2 weeks prior to initiation of CPI-613 treatment |  | |  |
|  | Documentation that the patient has not received cancer immunotherapy within the past 2 weeks prior to initiation of CPI-613 treatment |  | |  |
|  | Documentation that the patient does not have a serious medical illness that would potentially increase his or her risk for toxicity |  | |  |
|  | Documentation that the patient does not have any active uncontrolled bleeding or bleeding diathesis |  | |  |
|  | Documentation that the patient does not have an unrelated central nervous system (CNS) or epidural tumor |  | |  |
|  | Documentation that the patient is free of any condition or abnormality which may, in the opinion of the investigator, compromise his or her safety |  | |  |
|  | Documentation that the patient does not have active heart disease (including myocardial infarction within previous 6 months, symptomatic coronary artery disease, arrhythmias not controlled with medication, or symptomatic congestive heart failure) |  | |  |
|  | Documentation that the patient does not have a history of additional risk factors for torsade de pointes (e.g., heart failure, hypokalemia, family history of Long QT Syndrome) |  | |  |
|  | Documentation that the patient does not have evidence of active infection, or serious infection (e.g septic shock with multi-organ dysfunction) within the past month |  | |  |
|  | Documentation that the patient does not have known HIV infection |  | |  |
|  | Documentation that the patient does not require immediate palliative treatment of any kind, including surgery |  | |  |

# APPENDIX B STRC SAE REPORTING GUIDELINES

**Mandatory Safety and Toxicity Review Committee (STRC; Previously CROC)**

**Serious Adverse Event (SAE) Notification Procedure**

**Mandatory STRC SAE Reporting Requirements – Revised 6/05/2012**

This document describes STRC reporting and use of the electronic submission form that is submitted **for unexpected grade 4 and any grade 5 (death during protocol intervention) SAEs on CCCWFU Institutional interventional trial patients**. There are multiple entities that require reporting of SAEs. Each entity has different rules for what is reported, and how it is reported.

Rules used by other entities (Institutional Review Board (IRB), AdEERS, MedWatch, etc.) should NOT be used to evaluate whether an event should be reported to STRC. Only the rules for reporting described in this document should be considered.

As defined in the NCI summary IV reporting guidelines, **CCCWFU Institutional studies covered by these reporting requirements are defined as: In-house, internally reviewed trials, including those collaborative studies conducted with industry sponsorship in which the center is a primary contributor to the design, implementation, and monitoring of the trial, or participation in a multi-site trial initiated by an institutional investigator at another center.** Institutional trials are almost always authored by a researcher here at CCCWFU. Institutional protocols are labeled NCI Code=”I” for Institutional on the protocol screen in ORIS. Cooperative group protocols are **not** considered Institutional, but Research Base trials **are** classified as Institutional.

The STRC is responsible for reviewing SAEs for CCCWFU Institutional studies, as defined above. STRC currently requires that unexpected grade 4 and all grade 5 SAEs on these trials be reported to them for review. All Clinical Research Management (CRM) staff members assisting a PI in documenting and reporting an SAE that qualifies for STRC reporting are responsible for informing a clinical member of the STRC by phone, followed by informing the entire committee via the required email notification.

**THESE REPORTING REQUIREMENTS APPLY TO EVERYONE WORKING WITH CANCER CENTER INSTITUTIONAL PROTOCOLS.**

**What is considered an SAE under this mandatory procedure?**

Any **unexpected grade 4** event not including routinely experienced events per protocol (e.g. myelosuppression) and **all grade 5 events** (death during protocol intervention) should be reported. The patient is considered “on-treatment” as defined in the protocol, which can extend days/weeks/months past the last date of actual protocol intervention.

Top of Form

Bottom of Form

| **Table 1: Summary of STRC Reporting Requirements for Institutional Pilot, Phase 1, Phase 2 and Phase 3 Interventional Trials** | | | | | | |
| --- | --- | --- | --- | --- | --- | --- |
|  | **ADVERSE EVENT** | | | | | |
|  | **Grade 1, Grade 2, Grade 3** | | **Grade 4** | | **Grade 5** | |
|  | **Unexpected** | **Expected** | **Unexpected** | **Expected** | **Unexpected** | **Expected** |
| **Unrelated** | Not Required | Not Required | **REPORT TO STRC** | Not Required | **REPORT TO STRC** | **REPORT TO STRC** |
| **Unlikely** | Not Required | Not Required | **REPORT TO STRC** | Not Required | **REPORT TO STRC** | **REPORT TO STRC** |
| **Possible** | Not Required | Not Required | **REPORT TO STRC** | Not Required | **REPORT TO STRC** | **REPORT TO STRC** |
| **Probable** | Not Required | Not Required | **REPORT TO STRC** | Not Required | **REPORT TO STRC** | **REPORT TO STRC** |
| **Definite** | Not Required | Not Required | **REPORT TO STRC** | Not Required | **REPORT TO STRC** | **REPORT TO STRC** |

STRC reporting may not be appropriate for specific expected adverse events for protocols. In those situations the adverse events that will not require STRC reporting **must be specified in the text of the approved protocol.**

**STRC notification responsibilities of the person handling the reporting/documenting of the SAE:**

1. Make a phone call to the appropriate clinical member of the STRC as listed below (page if necessary)—see note 2 below
2. Submit the STRC Notification Form WITHIN 24 HOURS of first knowledge of the event. This form is found at either the ORIS main menu page or by going to <http://ccc.wfubmc.edu/oris/strc.aspx>.

This will ensure that all persons that the event applies to will be notified; remember to file a copy of your confirmation. (Form instructions will walk you through the required fields, consult the help page for further instructions.)

1. Ensure that you document that the appropriate persons on the STRC has been contacted.
2. Follow up with/update the clinical member of STRC regarding any new developments or information obtained during the course of the SAE investigation and reporting process.

**Elements needed to complete the electronic STRC form:**

1. ORIS Patient ID (PID)
2. Name of STRC Clinician notified/Date/Time/Comments.
3. Grade of event.
4. Is this related to protocol intervention or treatment?
5. Is suspension of the protocol needed?
6. Is any change to consent or protocol needed?
7. Was the nature or severity of the event unexpected?
8. Date of the event.
9. Brief description of the event using approved CTC version terminology.
10. Date of last study dose before event.
11. Relevant tests/labs.
12. **Most importantly make sure that the Investigator assigns attribution to the reported event (grade) using the appropriate CTCAE version for the protocol.**

**The Clinical Members of STRC to Notify by Phone or Page:**

**Bayard Powell, MD** – Director-at-Large, CCCWFU; Chair, PRC; Section Head, Hematology/Oncology. 6-7970 / 6-2701 / Pager 806-9308

**Glenn Lesser, MD** – Hematology Oncology 6-9527 / 6-0256 / Pager 806-8397

**Kathryn Greven, MD** – Vice Chair – Radiation Oncology. 3-3600 / Pager 806-8314

**Marissa Howard-McNatt, MD** – General Surgery 6-0545 / 806-6438

**Definition of Unavailable:** As a general guideline if the first clinician that is contacted does not respond to the phone call or page within a reasonable amount of time, then initiate contact with their backup. Give the back-up a reasonable amount of time to respond to a phone call or page before contacting another member. This is a general guideline. You must use your best judgment as a clinical research professional given the time of day, severity of the SAE, and other circumstances as to when it is appropriate to contact backup clinicians. If the event occurs near the end of day, then leave messages (voice or email) as appropriate and proceed with submitting your STRC notification form. The important criteria is that have taken reasonable steps to notify and document that you have initiated some type of contact to one or more of the clinical members of STRC.

**STRC CLINICAN RESPONSIBILITY:**

It is the responsibility of the STRC clinician to review all reported events, evaluate the events as they are reported; and communicate a response to the Investigator, event reporter and the members of STRC. The review will include but not be limited to the information reported; there may be times when additional information is needed in order for an assessment to be made further communication directly with the investigator may be warranted. STRC reserves the right to agree with the investigator’s assessment if STRC does not agree with the investigator STRC reserves the right to suspend the trial pending further investigation.

**AMENDMENTS TO PREVIOUS REPORTS**

If you are not able to supply all pertinent information with the initial submission, once the additional information is available **do not submit a new report**. Go to the original email that was received by STRC and others “reply to all” and entitle your email “**Amendment** for (list date of event and patient ID) this will avoid duplications of the same event. List the additional information which you are reporting.

| **Adverse Event Description** | **Cycle of Toxicity Onset** | **Start Date** | **Stop Date** | **AE Type** | **Grade (1-5)**  **per CTC v. 4.0** | **Attribution** | **Serious** | **Action Taken** | **MD initials / date** |
| --- | --- | --- | --- | --- | --- | --- | --- | --- | --- |
|  |  |  |  | 🞎Expected  🞎Unexpected | 🞎Mild/1  🞎Moderate/2  🞎Severe/3  🞎Life- threatening/4  🞎Death/5 | 🞎Related  🞎Probably  🞎Possible  🞎Unlikely  🞎Unrelated | 🞎No  🞎Hospitalization  🞎Disability  🞎Birth Defect  🞎Life-threatening  🞎Death  🞎Other: _______ | 🞎None  🞎Therapy Withheld  🞎Therapy D/C  🞎Therapy Adjusted  🞎Other: _______  🞎N/A |  |
|  |  |  |  | 🞎Expected  🞎Unexpected | 🞎Mild/1  🞎Moderate/2  🞎Severe/3  🞎Life- threatening/4  🞎Death/5 | 🞎Related  🞎Probably  🞎Possible  🞎Unlikely  🞎Unrelated | 🞎No  🞎Hospitalization  🞎Disability  🞎Birth Defect  🞎Life-threatening  🞎Death  🞎Other: _______ | 🞎None  🞎Therapy Withheld  🞎Therapy D/C  🞎Therapy Adjusted  🞎Other: _______  🞎N/A |  |
|  |  |  |  | 🞎Expected  🞎Unexpected | 🞎Mild/1  🞎Moderate/2  🞎Severe/3  🞎Life- threatening/4  🞎Death/5 | 🞎Related  🞎Probably  🞎Possible  🞎Unlikely  🞎Unrelated | 🞎No  🞎Hospitalization  🞎Disability  🞎Birth Defect  🞎Life-threatening  🞎Death  🞎Other: _______ | 🞎None  🞎Therapy Withheld  🞎Therapy D/C  🞎Therapy Adjusted  🞎Other: _______  🞎N/A |  |
|  |  |  |  | 🞎Expected  🞎Unexpected | 🞎Mild/1  🞎Moderate/2  🞎Severe/3  🞎Life- threatening/4  🞎Death/5 | 🞎Related  🞎Probably  🞎Possible  🞎Unlikely  🞎Unrelated | 🞎No  🞎Hospitalization  🞎Disability  🞎Birth Defect  🞎Life-threatening  🞎Death  🞎Other: _______ | 🞎None  🞎Therapy Withheld  🞎Therapy D/C  🞎Therapy Adjusted  🞎Other: _______  🞎N/A |  |

# APPENDIX C – CCCWFU ADVERSE EVENT LOG

# *Only Grade 3 OR higher Need to be recorded*

# APPENDIX D – Data Collection Form for Efficacy Assessment

**ORIS PID:** _______________________ **Date Completed:** ____ / ____ / ____

**Visit:**  Pre-study  Cycle 1 Visit  Cycle 2 Visit  Cycle 3 Visit  Cycle 4 Visit

Cycle 5 Visit  Cycle 6 Visit  Cycle 7 Visit  Cycle 8 Visit  Cycle 9 Visit

Cycle 10 Visit  Other (Specify: _________________________________)

**ECOG Performance Status (Date Performed:** ____ / ____ / ____)

0 Fully active, able to carry on all pre-disease performance without restriction.

1 Restricted in physically strenuous activity but ambulatory and able to carry out work of a light or sedentary nature, e.g., light house work, office work.

2 Ambulatory and capable of all self-care but unable to carry out any work activities. Up and about more than 50% of waking hours.

3 Capable of only limited self-care. Confined to bed or chair more than 50% of waking hours.

4 Completely disabled. Cannot carry on any self-care. Totally confined to bed or chair.

**Date of contrast CT (chest / abdomen) and/or PET/CT**: ____ / ____ / ____

**Were new lesions identified on contrast CT (chest / abdomen) and/or PET/CT?**  Yes  No

**Has this subject progressed according to RECIST criteria? (As determined by the treating physician)**  Yes  No

**If yes, please comment: ____________________________________________________________________________**

**____________________________________________________________________________**

**____________________________________________________________________________**

**____________________________________________________________________________**

**LDH (Day 1 of each cycle): __________ Date: ___ ___ / ___ ___ / ___ ___**

# APPENDIX E – Data collection form for telephone follow-up

This form is to be completed post-study, bi-monthly for each participant

**ORIS PID** _____________________ **Date of Bimonthly Telephone Call** ____ / ____ / ____

**Name of person completing form** __________________________

**What was the result of the telephone call?**

Call was unanswered

Spoke directly with the subject

Spoke to a family member

Left a message

Comments: ________________________________________________________________________________________________________________________________________________________

**Is this subject still living?** Yes  No  Unknown

**If the subject has expired, list date of death:** ____ / ____ / ____

**Is the subject currently receiving or has received since coming off the 62113 trial systemic chemotherapeutic cancer treatment?** Yes  No  Unknown

Type of cancer treatment being received (including the dose, dosing schedule and duration, if applicable and if known): _________________________________________________________________________

**Is the subject receiving radiation for cancer treatment?** Yes  No  Unknown

Type of radiation treatment being received: _________________________________________________________________________

**Is the subject receiving palliative / Hospice care?** Yes  No  Unknown

If yes, date of enrollment into Hospice: ___ ___ / ___ ___ / ___ ___

**COMMENTS: ________________________________________________________________**

**____________________________________________________________________________**

**____________________________________________________________________________**

# APPENDIX F – Withdrawal of participation data collection form

**ORIS PID** _______________________ **Date Completed** ____ / ____ / ____

**Name of person completing form** __________________________

**Did the subject meet eligibility criteria for study enrollment?** Yes  No

**Reasons for withdrawal: (*Check all that apply and provide additional information*)**

Patient exhibited progression of disease

Unacceptable toxicity from CPI-613

Patient withdrawal of consent

For just the CPI-613 administration only

For all components of the research study (including follow up in the medical record)

Investigator’s discretion to withdraw patient from the study because continued participation in the study is not in the patient’s best interest

Undercurrent illness: a condition, injury, or disease unrelated to the intended disease for which the study is investigating, that renders continuing the treatment unsafe or regular follow-up impossible

General or specific changes in the patient’s condition that renders the patient ineligible for further investigational treatment

Non-compliance with investigational treatment, protocol-required evaluations or follow-up visits

Termination of the clinical trial by the clinical sponsor

**COMMENTS: ________________________________________________________________**

**____________________________________________________________________________**

**____________________________________________________________________________**

**APPENDIX G – Measurement Form**

**Instructions:** Complete and submit this form as required by the protocol. Do not leave any entries blank. Enter -1 to indicate that an answer is unknown, unobtainable, not applicable or not done. Retain a copy for your records and submit original to the CCCWFU Data Management Center.

CCCWFU Study Number: __________________________ CCCWFU Patient ID: ________________________________

Patient Name: ____________________________________ MRN: ___________________________________

WFUHS/Affiliate: ____________________________________________________________________________________________________

**(Specify)**

| Date of Observation (mm/dd/yy) | |  |  |  |  |  |  |
| --- | --- | --- | --- | --- | --- | --- | --- |
| Cycle: | |  |  |  |  |  |  |
| **TARGET LESIONS** | |  |  |  |  |  |  |
| Response Status (CR, PR, SD, PD) | |  |  |  |  |  |  |
| List sites for response: | Means of Evaluation | Measurement | Measurement | Measurement | Measurement | Measurement | Measurement |
| 1. |  |  |  |  |  |  |  |
| 2. |  |  |  |  |  |  |  |
| 3. |  |  |  |  |  |  |  |
| 4. |  |  |  |  |  |  |  |
| 5. |  |  |  |  |  |  |  |
| 6. |  |  |  |  |  |  |  |
| 7. |  |  |  |  |  |  |  |
| 8. |  |  |  |  |  |  |  |
| 9. |  |  |  |  |  |  |  |
| 10. |  |  |  |  |  |  |  |
| Total sum of LD for all Target Lesions: | |  |  |  |  |  |  |
| **NON-TARGET LESIONS** | | | | | | | |
| Response Status  (CR, Incomplete Response/SD, PD): | |  |  |  |  |  |  |
| List sites for response | Means of Evaluation | Measurement | Measurement | Measurement | Measurement | Measurement | Measurement |
| 1. |  |  |  |  |  |  |  |
| 2. |  |  |  |  |  |  |  |
| 3. |  |  |  |  |  |  |  |
| 4. |  |  |  |  |  |  |  |
| 5. |  |  |  |  |  |  |  |
| 6. |  |  |  |  |  |  |  |
| 7. |  |  |  |  |  |  |  |
| **Observer Signature:** | |  | | | | | |

**LIST ALL TARGET AND NON-TARGET SITES TO BE USED FOR RESPONSE:**
